# Incremental prognostic value of pan-immune-inflammation value in conservatively treated acute myocardial infarction: derivation and MIMIC-IV validation

**DOI:** 10.3389/fcvm.2026.1935766

**Published:** 2026-09-10

**Authors:** Weilong Qiu, Huanhuan Yu, Xi Chen, Yuanyun Luo, Fujin Lin, Qingqing Luo, Jimin Fan, Yun Lin

**Affiliations:** 1 Department of Cardiology, Longyan First Affiliated Hospital of Fujian Medical University, Longyan City, Fujian, China; 2 Department of Neurosurgery, Sichuan Zigong Fourth People’s Hospital, Zigong City, Sichuan, China; 3 Department of Sleep Medicine, Second Affiliated Hospital of Fujian Medical University, Quanzhou City, Fujian, China

**Keywords:** acute myocardial infarction, all-cause mortality, immune-inflammatory indices, pan-Immune-Inflammation value, systemic immune-inflammation index, systemic inflammation response index

## Abstract

**Introduction:**

Immune-inflammatory activation contributes to adverse outcomes after acute myocardial infarction (AMI). the study evaluated whether the pan-immune-inflammation value (PIV) was associated with 6-month all-cause mortality and provided prognostic information beyond conventional clinical predictors.

**Methods:**

This retrospective two-cohort study included 2,008 conservatively treated patients with AMI in the derivation cohort and 2,679 patients with AMI from the Medical Information Mart for Intensive Care IV (MIMIC-IV) in an independent evaluation cohort. PIV was prespecified as the primary index, with the systemic inflammation response index (SIRI) and systemic immune-inflammation index (SII) assessed as complementary indices. Associations were examined using multivariable logistic regression. Incremental performance was assessed against a prespecified clinical model, with 1,000-resample bootstrap internal validation and Firth penalized logistic regression. Owing to differences in variable availability, MIMIC-IV analyses used a cohort-specific adjustment model.

**Results:**

Within 6 months, 57 patients died in the derivation cohort and 773 died in MIMIC-IV. In the derivation cohort, each 1-standard-deviation increase in log-transformed PIV was independently associated with mortality (odds ratio [OR], 1.56; 95% confidence interval [CI], 1.19–2.04; *P* = 0.001), with a similar Firth estimate (OR, 1.55; 95% CI, 1.19-2.01; *P* = 0.001). Adding PIV increased the area under the receiver operating characteristic curve from 0.844 to 0.854 (Δ = 0.010; *P* = 0.169); the corresponding optimism-corrected values were 0.813 and 0.823 (corrected Δ = 0.0106). SIRI and SII yielded supportive findings. In MIMIC-IV, PIV remained associated with 6-month mortality (OR, 1.20; 95% CI, 1.10–1.32; *P* < 0.001), while the area under the curve increased only from 0.770 to 0.775 after adding PIV.

**Conclusion:**

Higher PIV was independently associated with mortality after AMI across both cohorts, but its incremental discrimination beyond conventional clinical factors was modest and statistically nonsignificant. The MIMIC-IV findings support the reproducibility of the association rather than direct validation of an unchanged prediction model. PIV may therefore be considered a complementary marker rather than a stand-alone risk-stratification tool. Prospective multicenter studies are required before clinical implementation.

## Introduction

Acute myocardial infarction (AMI) remains a major cause of cardiovascular morbidity and mortality worldwide despite substantial advances in diagnostic strategies, pharmacological therapy, and revascularization techniques (1, 2). Current definitions and management guidelines emphasize early diagnosis, timely risk stratification, and individualized treatment allocation, because patients with AMI present with markedly heterogeneous clinical profiles and long-term outcomes (3). Although reperfusion therapy has improved survival in many patients, a considerable proportion of patients are still managed conservatively because of delayed presentation, high procedural risk, advanced comorbidity burden, or local treatment constraints. For these patients, identifying simple and reproducible prognostic indicators that can be obtained early during hospitalization remains clinically important.

Inflammation and immune activation are central to the development and progression of atherosclerosis and play important roles in plaque destabilization, coronary thrombosis, myocardial injury, and post-infarction repair (4, 5). After AMI, neutrophils, monocytes/macrophages, lymphocytes, platelets, and endothelial cells participate dynamically in ischemic injury, infarct healing, ventricular remodeling, and systemic complications (6, 7). Clinical trials targeting inflammatory pathways, including interleukin-1β inhibition and colchicine therapy, have further supported the concept that residual inflammatory risk is not merely a bystander phenomenon but may influence cardiovascular outcomes after atherosclerotic events and myocardial infarction (8, 9). Therefore, immune-inflammatory status may provide prognostic information beyond traditional clinical variables in AMI.

Complete blood count-derived inflammatory indices have attracted increasing attention because they are inexpensive, routinely available, and biologically interpretable. Traditional markers such as the neutrophil-to-lymphocyte ratio (NLR) reflect the balance between innate inflammatory activation and adaptive immune regulation and have been associated with adverse atherosclerotic outcomes (10, 11). More recently, composite immune-inflammatory indices, including the pan-immune-inflammation value (PIV), systemic inflammation response index (SIRI), and systemic immune-inflammation index (SII), have been proposed to integrate multiple circulating immune and thrombotic components. PIV incorporates neutrophils, monocytes, platelets, and lymphocytes, thereby capturing inflammatory activation, platelet-related thrombosis, monocyte-mediated vascular injury, and lymphocyte-associated immune regulation in a single metric. Compared with single blood-cell parameters, such composite indices may better reflect the coordinated immune-thrombotic response involved in AMI progression.

However, several knowledge gaps remain. First, most previous AMI risk stratification approaches have focused on demographic characteristics, hemodynamic status, cardiac functional severity, comorbidities, and conventional biomarkers, whereas the incremental value of immune-inflammatory indices beyond a prespecified clinical model remains insufficiently clarified. Second, available inflammatory markers are often evaluated individually, and few studies have compared PIV with related composite indices such as SIRI and SII within the same analytical framework. Third, external validation is essential before a biomarker can be considered robust, yet many single-center studies lack independent validation in real-world electronic health record databases. The Medical Information Mart for Intensive Care IV (MIMIC-IV) provides a large, de-identified, structured critical care database that can support external validation and assessment of generalizability across different healthcare settings (12). At the same time, clinical prediction research requires transparent model construction and careful evaluation of discrimination, calibration, clinical utility, and incremental predictive performance (13).

In this study, which investigated the association between PIV and 6-month all-cause mortality in a retrospective derivation cohort of conservatively treated patients with AMI. SIRI and SII were evaluated as complementary immune-inflammatory indices. The research further examined whether adding these indices improved prognostic performance beyond a prespecified reference clinical model constructed from routinely available conventional predictors. To assess robustness and transportability, moreover it externally validated the findings in a broader real-world AMI cohort from MIMIC-IV and performed sensitivity analyses using alternative mortality endpoints. The present study hypothesized that higher systemic immune-inflammatory burden, particularly reflected by PIV, would be independently associated with mortality after AMI and would provide modest but clinically meaningful incremental prognostic information beyond conventional clinical variables.

## Materials and methods

### Study design and ethical approval

This retrospective observational study included a derivation cohort and an independent MIMIC-IV external validation cohort. Longyan First Affiliated Hospital of Fujian Medical University was the coordinating institution, and the study was approved by its Ethics Committee (Approval No. LYREC2025-k104-01). Data for the derivation cohort were provided by Sichuan Zigong Fourth People's Hospital under local ethical approval (Approval No. 2020-010). The requirement for written informed consent was waived because of the retrospective and de-identified nature of the study. The study was conducted in accordance with the Declaration of Helsinki. The MIMIC-IV v2.2 database contains de-identified clinical data and was accessed through PhysioNet by credentialed investigators after completion of the required human-subjects research training and approval of the PhysioNet data-use agreement.

### Derivation cohort

**Figure 1:**
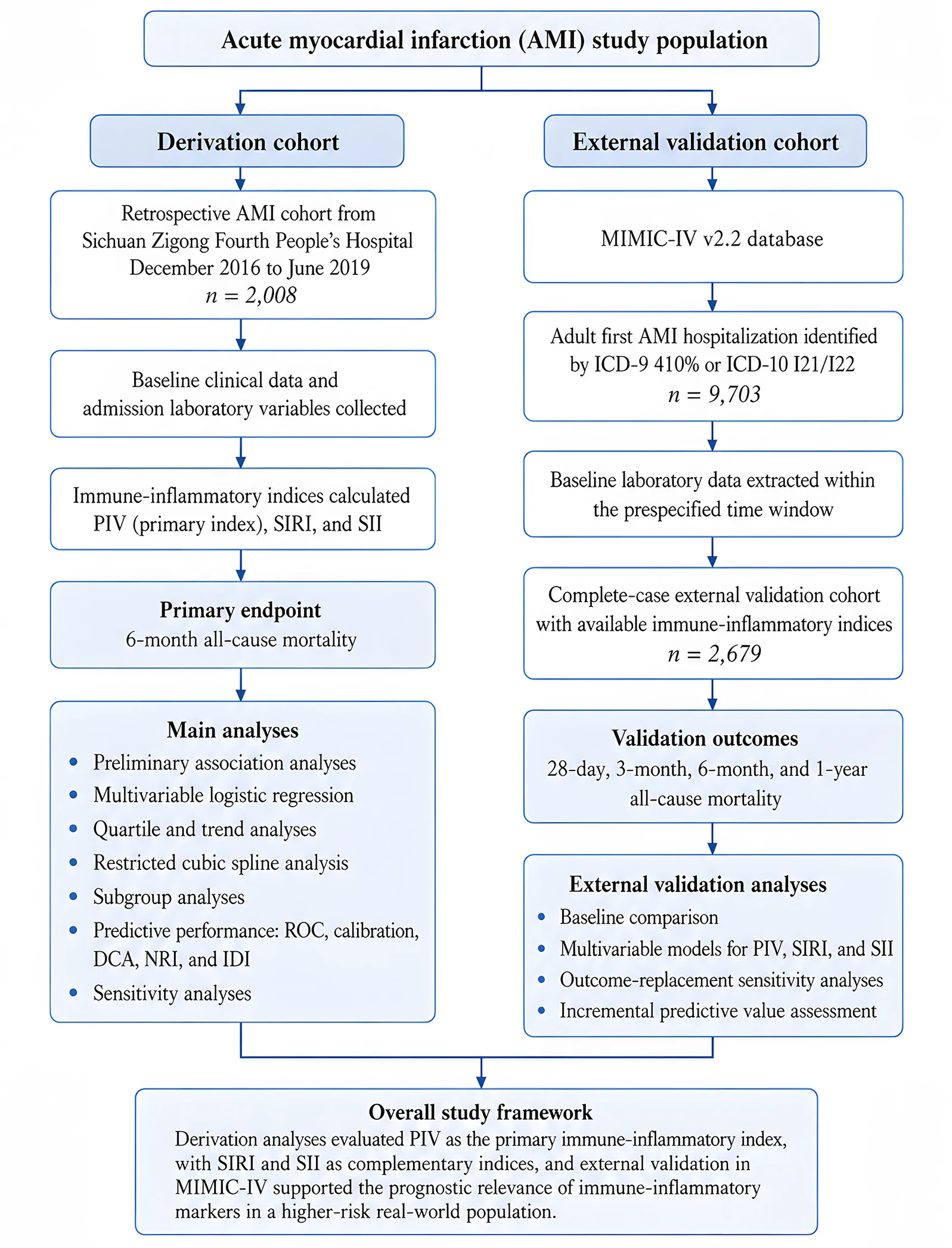
Study flowchart of the derivation cohort and MIMIC-IV external validation cohort. The derivation cohort included 2,008 conservatively treated patients with acute myocardial infarction (AMI) from Sichuan Zigong Fourth People's Hospital between December 2016 and June 2019. Baseline clinical and laboratory data were collected to calculate the pan-immune-inflammation value (PIV), systemic inflammation response index (SIRI), and systemic immune-inflammation index (SII). The primary endpoint was 6-month all-cause mortality. In the external validation cohort, adult first AMI hospitalizations were identified from MIMIC-IV v2.2 using ICD-9 codes beginning with 410 or ICD-10 codes beginning with I21/I22. After complete-case selection based on available immune-inflammatory indices, 2,679 patients were included for external validation. ROC, receiver operating characteristic; DCA; NRI, net reclassification improvement; IDI, integrated discrimination improvement.

Consecutive patients hospitalized with AMI at Sichuan Zigong Fourth People's Hospital between December 2016 and June 2019 were retrospectively assessed. AMI was diagnosed according to compatible clinical symptoms, electrocardiographic findings, and cardiac biomarker elevation. The source cohort consisted of patients managed medically without reperfusion therapy. The inclusion criteria were: 1) Diagnosis of AMI during the study period; 2) Age ≥18 years; 3) Conservative medical treatment without percutaneous coronary intervention, coronary artery bypass grafting, or thrombolysis during the index hospitalization; 4) Availability of admission clinical and laboratory data required to calculate the immune-inflammatory indices; and 5) Availability of 6-month vital-status information. The exclusion criteria were: 1) Age <18 years or missing age information; 2) Receipt of reperfusion therapy; 3) Duplicate admissions, with only the first eligible hospitalization retained; 4) Hospital stay <24 h; 5) Missing neutrophil, lymphocyte, monocyte, or platelet measurements; 6) Missing covariates required for the prespecified analyses; and 7) Unavailable follow-up mortality data. A total of 2,008 patients were included in the derivation cohort (Figure 1).

### MIMIC-IV external validation cohort

Data for the external validation cohort were obtained from MIMIC-IV v2.2 through PhysioNet (12, 14). MIMIC-IV is a de-identified electronic health record database developed by the Massachusetts Institute of Technology Laboratory for Computational Physiology and Beth Israel Deaconess Medical Center. Access was granted to credentialed investigators after completion of the required human-subjects research training and approval of the PhysioNet data-use agreement. Data extraction was performed from the MIMIC-IV hosp and icu modules using structured tables, including patients, admissions, diagnoses_icd, d_icd_diagnoses, icustays, labevents, and d_labitems. De-identified subject_id and hadm_id fields were used to link patient-level, admission-level, diagnosis, ICU, and laboratory information. Adult AMI hospitalizations were identified in MIMIC-IV v2.2 using International Classification of Diseases, Ninth Revision, Clinical Modification (ICD-9-CM) codes beginning with 410 or International Classification of Diseases, Tenth Revision, Clinical Modification (ICD-10-CM) codes beginning with I21 or I22. For patients with multiple eligible admissions, only the first AMI hospitalization was retained, yielding 9,703 adults for initial screening. The inclusion criteria were: 1) Age ≥18 years at admission; 2) First hospitalization with an eligible AMI diagnosis code; 3) Valid linked hospital admission data; and 4) Availability of baseline neutrophil, lymphocyte, monocyte, and platelet values within 6 h before to 24 h after admission, either directly measured or obtained using the prespecified derivation algorithm. The exclusion criteria were: 1) Subsequent eligible AMI admissions from the same patient; 2) Missing or nonpositive values required to calculate PIV, SIRI, or SII; and 3) Duplicate or invalid subject–admission records. After complete-case selection, 2,679 patients comprised the external validation cohort (Figure 1). Reperfusion status was not used as an eligibility criterion in MIMIC-IV because treatment status could not be defined using a validated structured algorithm directly comparable with that applied in the derivation cohort. Therefore, MIMIC-IV represented a broader real-world AMI population rather than an exact replication of the conservatively treated derivation cohort. The cohorts also differed in clinical setting, baseline risk, treatment patterns, and variable availability. In particular, Killip class, New York Heart Association (NYHA) class, symptom-to-door time, and heart failure (HF) type were not available as directly comparable structured variables in MIMIC-IV. Consequently, the external analysis used a cohort-specific clinical model based on available demographic, admission-related, and comorbidity variables. Thus, the MIMIC-IV analysis was intended primarily to evaluate the transportability of the associations and incremental predictive patterns rather than to validate an identical prediction model across the two cohorts.

### Data collection and immune-inflammatory indices

For the derivation cohort, demographic characteristics, cardiac functional status, medical history, comorbidities, and admission laboratory results were obtained from electronic medical records. Age was analyzed using the original ordered categories. HF type was classified as right-sided, left-sided, or both-sided heart failure, and both-sided heart failure was entered as a binary variable in the fully adjusted association model. Symptom-to-door time was retained as the original ordered variable.

For MIMIC-IV, demographic characteristics, race, insurance, admission type, intensive care unit admission, Charlson comorbidity index, comorbidities, and laboratory results were extracted from structured tables. Laboratory values obtained from 6 h before to 24 h after admission were screened, and the valid positive measurement closest to admission was retained. Absolute leukocyte subtype counts were preferentially used. When unavailable in MIMIC-IV, absolute counts were estimated from the white blood cell count and corresponding differential percentage. The indices were calculated as follows: PIV: Neutrophil count×platelet count×monocyte count/lymphocyte count; SIRI: Neutrophil count×monocyte count/lymphocyte count; SII: Platelet count×neutrophil count/lymphocyte count. PIV was prespecified as the primary index, whereas SIRI and SII were evaluated as complementary indices. All indices were natural log-transformed and standardized before continuous-variable regression analyses.

### Outcome assessment

The primary outcome in the derivation cohort was 6-month all-cause mortality. The 28-day and 3-month mortality endpoints were examined in sensitivity analyses. In MIMIC-IV, mortality was determined from the recorded date of death relative to the index admission. The evaluated outcomes were 28-day, 3-month, 6-month, and 1-year all-cause mortality. Six-month mortality was defined as death within 180 days after admission.

### Statistical analysis

Continuous variables were reported as medians with interquartile ranges and compared using the Mann–Whitney U test. Categorical variables were reported as numbers and percentages and compared using the chi-square test or Fisher's exact test, as appropriate. Univariable logistic regression and receiver operating characteristic analysis were used to examine preliminary associations. Each index was also categorized into quartiles, with Q1 as the reference group.

Covariates were prespecified based on clinical relevance, routine availability at or near admission, and established associations with mortality after AMI; no outcome-driven stepwise selection was performed. In the derivation cohort, Model 1 was unadjusted; Model 2 included age category, sex, Killip class, NYHA class, and Charlson comorbidity index; and Model 3 additionally included symptom-to-door time, both-sided HF, previous myocardial infarction, chronic HF, diabetes mellitus (DM), and chronic kidney disease (CKD). Complete-case analysis was used without statistical imputation, as valid blood-cell measurements and complete prespecified covariate data were required; the resulting sample sizes are shown in the cohort flowchart. Spearman correlations were used to assess correlations among PIV, SIRI, and SII. Because these indices share blood-cell components and were strongly correlated, they were evaluated in separate models. Multicollinearity was further assessed using the variance inflation factor for PIV in the fully adjusted derivation-cohort model. A reference clinical model was constructed from routinely available conventional predictors, including age category, sex, Killip class, NYHA class, Charlson comorbidity index, previous myocardial infarction, chronic HF, DM, CKD, and acute renal failure. PIV, SIRI, and SII were then added separately to this model to evaluate their incremental predictive value. Model performance was evaluated using the AUROC for discrimination, Brier score for overall prediction error, and AIC for model fit. Smoothed calibration curves were used to assess agreement between predicted and observed risks. Incremental performance was evaluated using changes in AUROC, continuous net reclassification improvement, and integrated discrimination improvement. DCA compared the estimated net benefit of the clinical and index-augmented models across clinically relevant threshold probabilities. Because DCA does not demonstrate improvement in actual patient outcomes, its findings were interpreted as exploratory evidence of potential clinical utility. The reference clinical model was constructed *a priori* to provide a conventional comparator for evaluating the incremental prognostic value of immune-inflammatory indices. Candidate variables were selected according to clinical relevance, routine availability at or near admission, and established prognostic associations with mortality after AMI, rather than by data-driven variable selection. Details on the rationale for covariate selection and construction of the reference clinical models are provided in Supplementary Methods S1.

Because several derivation-cohort variables were not available as directly comparable structured variables in MIMIC-IV, the external validation models followed the same analytical principle but used MIMIC-IV-available covariates. For MIMIC-IV, Model 1 was unadjusted. Model 2 was adjusted for age, sex, race, admission type, and intensive care unit admission. Model 3 was further adjusted for insurance, Charlson comorbidity index, chronic HF, DM, CKD, acute renal failure, and history of myocardial infarction. External model performance and mortality associations across different follow-up intervals were evaluated using the same general analytical framework. Thus, the derivation and MIMIC-IV models followed the same construction principle, but the covariate set was adapted according to cohort-specific structured variable availability. The conceptual workflow and covariate-availability framework are shown in Supplementary Figure S1 and Supplementary Table S1. To assess potential overfitting, the reference clinical model and the PIV-augmented model underwent internal validation using 1,000 paired nonparametric bootstrap resamples. Both models were refitted in each resample, and optimism-corrected AUROC, Brier score, calibration slope, and calibration intercept were calculated by subtracting mean bootstrap optimism from the apparent estimates. Given the limited event count, Firth penalized logistic regression was additionally used as a small-sample sensitivity analysis of the fully adjusted continuous-PIV association, with profile penalized-likelihood 95% confidence intervals and penalized-likelihood ratio *P* values. Continuous NRI and IDI were considered exploratory and interpreted alongside *Δ*AUROC, bootstrap-corrected performance, calibration, Brier score, and DCA findings. Additional details are provided in Supplementary Methods S2. All tests were two-sided, and *P* < 0.05 was considered statistically significant. Analyses were performed using Python version 3.12.

## Results

### Study population and baseline characteristics

The study framework, including the derivation and MIMIC-IV external validation cohorts, is shown in Figure 1. The derivation cohort included 2,008 patients with AMI, of whom 57 died and 1,951 survived during the 6-month follow-up. Baseline characteristics are presented in Supplementary Table S1. Compared with survivors, non-survivors had more severe cardiac dysfunction, worse renal function, and higher levels of neutrophils, D-dimer, high-sensitivity cardiac troponin, and B-type natriuretic peptide. PIV, SIRI, and SII were significantly higher in non-survivors. Median PIV was 664.16 [Interquartile Range (IQR), 253.92–1,041.13] in non-survivors vs. 286.00 (IQR, 147.78–591.19) in survivors; corresponding values were 3.99 vs. 2.18 for SIRI and 1,394.12 vs. 694.01 for SII (all *P* < 0.001; Table 1).

**Table 1:** Preliminary associations of immune-inflammatory indices with 6-month all-cause mortality in patients with acute myocardial infarction.

Immune-inflammatory index	Survivors (*n* = 1951), median (IQR)	Non-survivors (*n* = 57), median (IQR)	*P* value for group comparison	AUROC (95% CI)	Crude OR per 1-SD increase (95% CI)	*P* value for OR	Q4 vs. Q1 crude OR (95% CI)	*P* for trend across quartiles
PIV	286.00 (147.78–591.19)	664.16 (253.92–1041.13)	<0.001	0.677 (0.608–0.741)	1.79 (1.39–2.30)	<0.001	10.57 (3.21–34.87)	<0.001
SIRI	2.18 (1.20–3.89)	3.99 (2.26–7.74)	<0.001	0.678 (0.610–0.743)	1.72 (1.35–2.18)	<0.001	10.20 (3.09–33.70)	<0.001
SII	694.01 (406.35–1267.92)	1394.12 (639.61–2310.32)	<0.001	0.666 (0.585–0.740)	1.79 (1.39–2.30)	<0.001	5.25 (2.17–12.74)	<0.001

Values are presented as median (interquartile range). Group comparisons were performed using the Mann–Whitney U test. Crude odds ratios were estimated using univariable logistic regression and are reported per 1-standard-deviation increase in the natural log-transformed index. Quartiles were generated according to the distribution of each immune-inflammatory index, with Q1 used as the reference group. The outcome was 6-month all-cause mortality. A two-sided *P* value <0.05 was considered statistically significant.

Formula definitions: PIV=neutrophil count x platelet count x monocyte count/lymphocyte count; SIRI=neutrophil count x monocyte count/lymphocyte count; SII=platelet count x neutrophil count/lymphocyte count.

AMI, acute myocardial infarction; AUROC, area under the receiver operating characteristic curve; CI, confidence interval; IQR, interquartile range; OR, odds ratio; SD, standard deviation; PIV, pan-immune-inflammation value; SII, systemic immune-inflammation index; SIRI, systemic inflammation response index.

### Associations of immune-inflammatory indices with 6-month mortality

**Figure 2:**
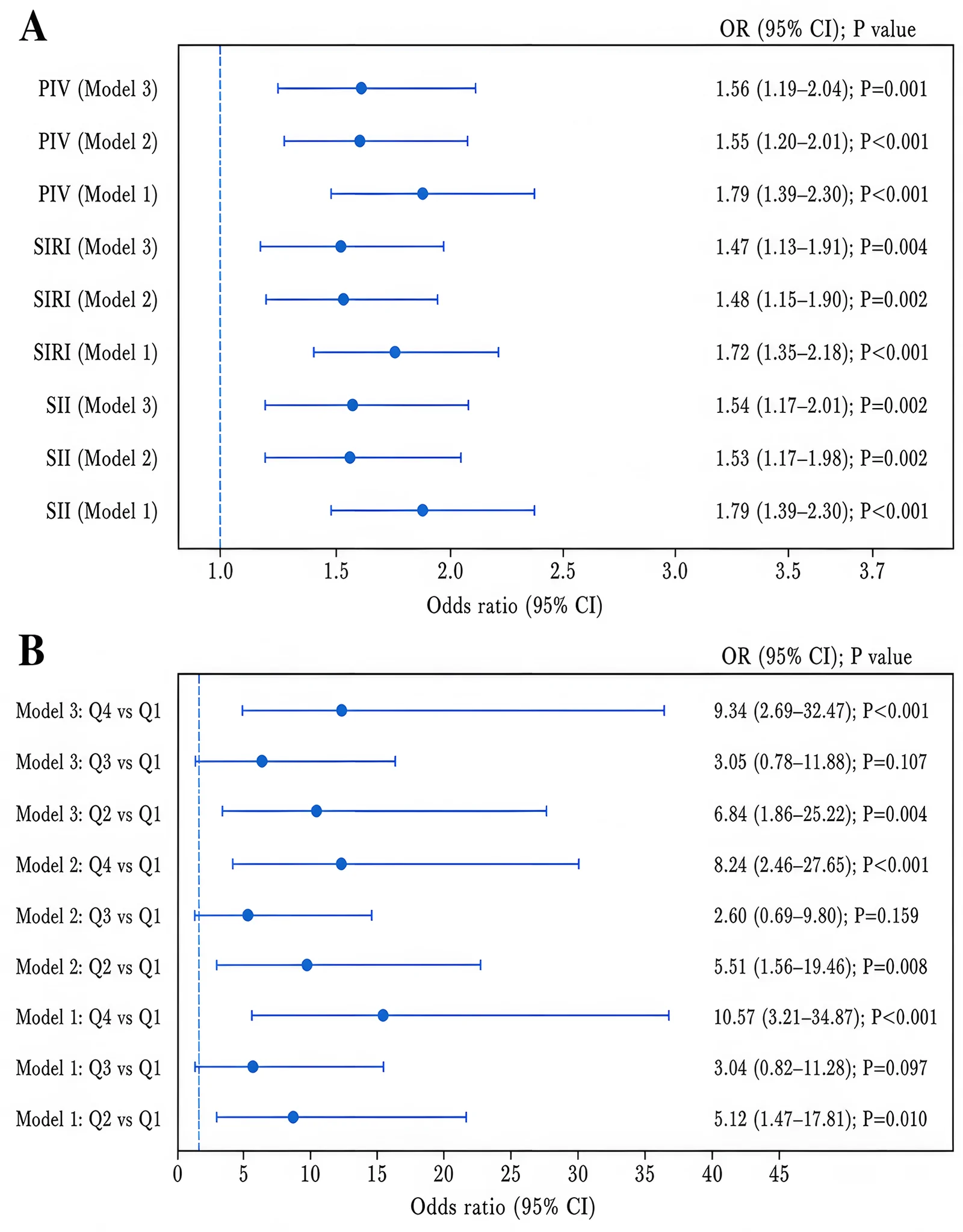
Multivariable and quartile analyses of immune-inflammatory indices for 6-month all-cause mortality in the derivation cohort. **(A)** Forest plot showing odds ratios (ORs) and 95% confidence intervals (CIs) for 6-month mortality per 1-standard-deviation increase in log-transformed PIV, SIRI, and SII across sequential regression models. Model 1 was unadjusted; Model 2 was adjusted for age category, sex, Killip class, New York Heart Association class, and Charlson comorbidity index; Model 3 was further adjusted for symptom-to-door time, both-sided heart failure, history of myocardial infarction, history of chronic heart failure, diabetes mellitus, and chronic kidney disease. **(B)** Quartile analysis of PIV, with Q1 as the reference group, across Models 1–3. The vertical dashed line indicates OR=1. PIV, pan-immune-inflammation value; SIRI, systemic inflammation response index; SII, systemic immune-inflammation index.

The preliminary associations are summarized in Table 1. PIV yielded an AUROC of 0.677 [95% Confidence Interval (95%CI), 0.608–0.741], and each one-standard-deviation (1-SD) increase in log-transformed PIV was associated with higher mortality in the univariable model [Odds Ratio (OR), 1.79; 95% CI, 1.39–2.30; *P* < 0.001]. Patients in the highest PIV quartile had a markedly higher risk than those in the lowest quartile (OR, 10.57; 95% CI, 3.21–34.87; P for trend <0.001). SIRI and SII showed similar associations, with AUROCs of 0.678 and 0.666, respectively. PIV remained independently associated with 6-month mortality after multivariable adjustment (Table 2 and Figure 2A). In the fully adjusted model, each 1-SD increase in log-transformed PIV was associated with a 56% increase in the odds of mortality (OR, 1.56; 95% CI, 1.19–2.04; *P* = 0.001). The AIC decreased from 502.1 in the unadjusted model to 428.5 in Model 3.

**Table 2:** Association of PIV with 6-month all-cause mortality in patients with acute myocardial infarction: primary continuous analysis and quartile-based sensitivity analysis.

Part A Primary analysis: PIV modeled as a continuous variable			
Model	OR (95% CI)	*P* value	AIC
Model 1	1.79 (1.39–2.30)	<0.001	502.1
Model 2	1.55 (1.20–2.01)	<0.001	450.4
Model 3	1.56 (1.19–2.04)	0.001	428.5

Part B Sensitivity analysis: PIV categorized into quartiles				
Model	Quartile	OR (95% CI)	*P* value	*P* for trend
Model 1	Q2 vs. Q1	5.12 (1.47–17.81)	0.010	<0.001
Model 1	Q3 vs. Q1	3.04 (0.82–11.28)	0.097	
Model 1	Q4 vs. Q1	10.57 (3.21–34.87)	<0.001	
Model 2	Q2 vs. Q1	5.51 (1.56–19.46)	0.008	<0.001
Model 2	Q3 vs. Q1	2.60 (0.69–9.80)	0.159	
Model 2	Q4 vs. Q1	8.24 (2.46–27.65)	<0.001	
Model 3	Q2 vs. Q1	6.84 (1.86–25.22)	0.004	<0.001
Model 3	Q3 vs. Q1	3.05 (0.78–11.88)	0.107	
Model 3	Q4 vs. Q1	9.34 (2.69–32.47)	<0.001	

**Notes:** Odds ratios in Part A are presented per 1-SD increase in natural log-transformed PIV. In Part B, PIV was categorized into quartiles, with Q1 as the reference group. Model 1 was unadjusted. Model 2 was adjusted for age category, sex, Killip class, NYHA class, symptom-to-door time, and HF type. Model 3 was further adjusted for Charlson comorbidity index, history of myocardial infarction, history of chronic heart failure, diabetes mellitus, chronic kidney disease, and acute renal failure. P for trend was calculated by entering PIV quartiles as an ordinal variable into the corresponding logistic regression model. HF type was treated as a nominal categorical variable.

**Abbreviations:** AIC, Akaike information criterion; CI, confidence interval; HF, heart failure; NYHA, New York Heart Association; OR, odds ratio; PIV, pan-immune-inflammation value; SD, standard deviation.

Quartile analysis also supported the association (Table 2 and Figure 2B). Compared with Q1, the fully adjusted ORs were 6.84 (95% CI, 1.86–25.22) for Q2, 3.05 (95% CI, 0.78–11.88) for Q3, and 9.34 (95% CI, 2.69–32.47) for Q4, with a significant ordinal trend (P for trend <0.001). However, the category-specific estimates were not strictly monotonic and had wide confidence intervals.

**Figure 3:**
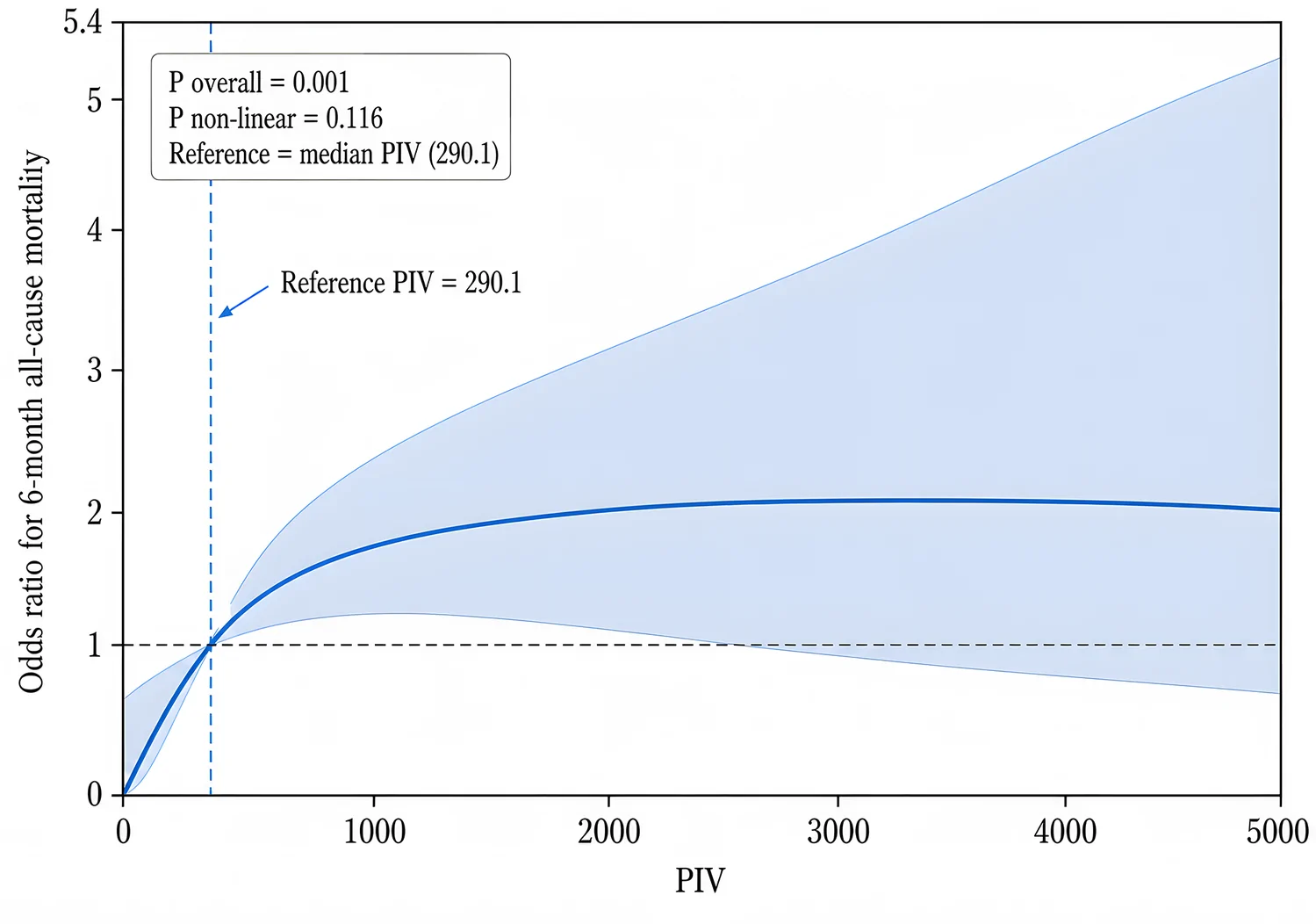
Restricted cubic spline analysis of PIV and 6-month all-cause mortality in the derivation cohort. The solid blue line represents the adjusted odds ratio for 6-month mortality across the continuous distribution of PIV, and the shaded area represents the 95% confidence interval. The vertical dashed line indicates the reference value, defined as the median PIV of 290.1, and the horizontal dashed line indicates an odds ratio of 1. The overall association was significant (P overall=0.001), without significant evidence of nonlinearity (P non-linear=0.116). PIV, pan-immune-inflammation value.

Restricted cubic spline (RCS) analysis showed a significant overall association between PIV and 6-month mortality (Figure 3; P overall = 0.001). Using the median PIV of 290.1 as the reference, mortality risk generally increased with PIV, without significant evidence of nonlinearity (P nonlinear=0.116).

### Incremental predictive value

**Figure 4:**
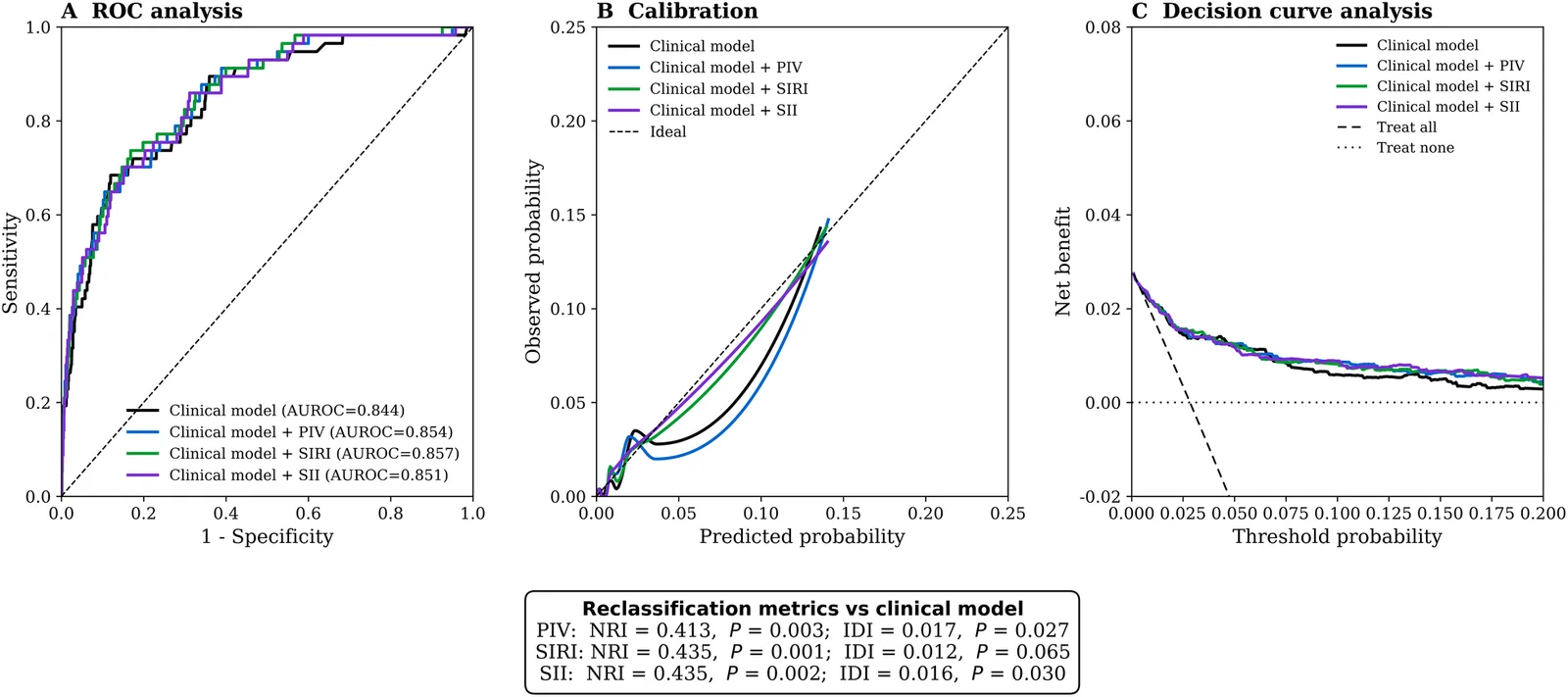
Predictive performance and clinical utility of immune-inflammatory indices for 6-month all-cause mortality in the derivation cohort. **(A)** Receiver operating characteristic curves comparing the clinical model alone with the clinical model augmented by pan-immune-inflammation value (PIV), systemic inflammation response index (SIRI), or systemic immune-inflammation index (SII). **(B)** Smoothed calibration curves of the clinical model and the three index-augmented models, showing the agreement between predicted and observed 6-month all-cause mortality. **(C)** Decision curve analysis comparing the net clinical benefit of the four models across a range of threshold probabilities. The boxed reclassification metrics summarize the incremental predictive value of each immune-inflammatory index relative to the clinical model alone. AUROC, area under the receiver operating characteristic curve; IDI, integrated discrimination improvement; NRI, net reclassification improvement.

The prespecified reference clinical model, constructed from routinely available conventional prognostic predictors as described in the Methods and Supplementary Methods S1, achieved an AUROC of 0.844 (95% CI, 0.786–0.894; Table 3 and Figure 4A). Adding PIV increased the AUROC to 0.854 (95% CI, 0.802–0.899; *Δ*AUROC = 0.010; *P* = 0.169), while the SIRI- and SII-extended models yielded AUROCs of 0.857 (95% CI, 0.803–0.905; *Δ*AUROC = 0.013; *P* = 0.051) and 0.851 (95% CI, 0.800–0.900; *Δ*AUROC = 0.007; *P* = 0.408), respectively. The PIV-, SIRI-, and SII-extended models showed slightly lower Brier scores and AIC values than the clinical model alone. Exploratory reclassification analysis yielded positive continuous NRI estimates for all three immune-inflammatory indices compared with the clinical model, with NRI values of 0.413 for PIV (*P* = 0.003), 0.435 for SIRI (*P* = 0.001), and 0.435 for SII (*P* = 0.002). IDI was significant for PIV (0.017; *P* = 0.027) and SII (0.016; *P* = 0.030), whereas the IDI improvement for SIRI did not reach statistical significance (0.012; *P* = 0.065). Smoothed calibration curves showed acceptable agreement between predicted and observed 6-month mortality across the clinical and index-augmented models (Figure 4B). DCA showed that the index-augmented models provided a modest net clinical benefit compared with the clinical model alone across a range of threshold probabilities (Figure 4C). In 1,000-resample bootstrap internal validation, the optimism-corrected AUROC was 0.813 for the reference clinical model and 0.823 for the PIV-augmented model, corresponding to a corrected increment of 0.0106. The optimism-corrected Brier scores were 0.0259 and 0.0255, respectively; the corresponding calibration slopes were 0.829 and 0.831, and the calibration intercepts were 0.010 and 0.008 (Supplementary Methods S2).

**Table 3:** Incremental predictive value of immune-inflammatory indices beyond the clinical model.

Model	AUROC (95% CI)	*Δ*AUROC	*P* for *Δ*AUROC	Brier score	AIC	NRI (95% CI)	*P* for NRI	IDI (95% CI)	*P* for IDI
Clinical model	0.844 (0.786–0.894)	Reference	Reference	0.025	432.3	Reference	Reference	Reference	Reference
Clinical model + PIV	0.854 (0.802–0.899)	0.010	0.169	0.024	423.2	0.413 (0.159–0.666)	0.003	0.017 (0.002–0.032)	0.027
Clinical model + SIRI	0.857 (0.803–0.905)	0.013	0.051	0.024	424.8	0.435 (0.174–0.681)	0.001	0.012 (−0.001–0.025)	0.065
Clinical model + SII	0.851 (0.800–0.900)	0.007	0.408	0.024	424.4	0.435 (0.176–0.699)	0.002	0.016 (0.001–0.030)	0.030

The clinical model included age category, sex, Killip class, New York Heart Association class, Charlson comorbidity index, history of myocardial infarction, history of chronic heart failure, diabetes mellitus, chronic kidney disease, and acute renal failure. PIV, SIRI, and SII were separately added to the clinical model after natural logarithmic transformation and standardization. Continuous NRI and IDI were calculated by comparing each extended model with the clinical model. These reclassification measures were considered exploratory and were interpreted together with the magnitude and statistical uncertainty of *Δ*AUROC. Bootstrap resampling was used to estimate 95% confidence intervals and *P* values. Optimism-corrected performance for the primary PIV model comparison is reported in Supplementary Methods S2.

AIC, Akaike information criterion; AUROC, area under the receiver operating characteristic curve; *Δ*AUROC, change in the AUROC; CI, confidence interval; IDI, integrated discrimination improvement; NRI, net reclassification improvement; NYHA, New York Heart Association; PIV, pan-immune-inflammation value; SII, systemic immune-inflammation index; SIRI, systemic inflammation response index.

### Subgroup and sensitivity analyses

**Figure 5:**
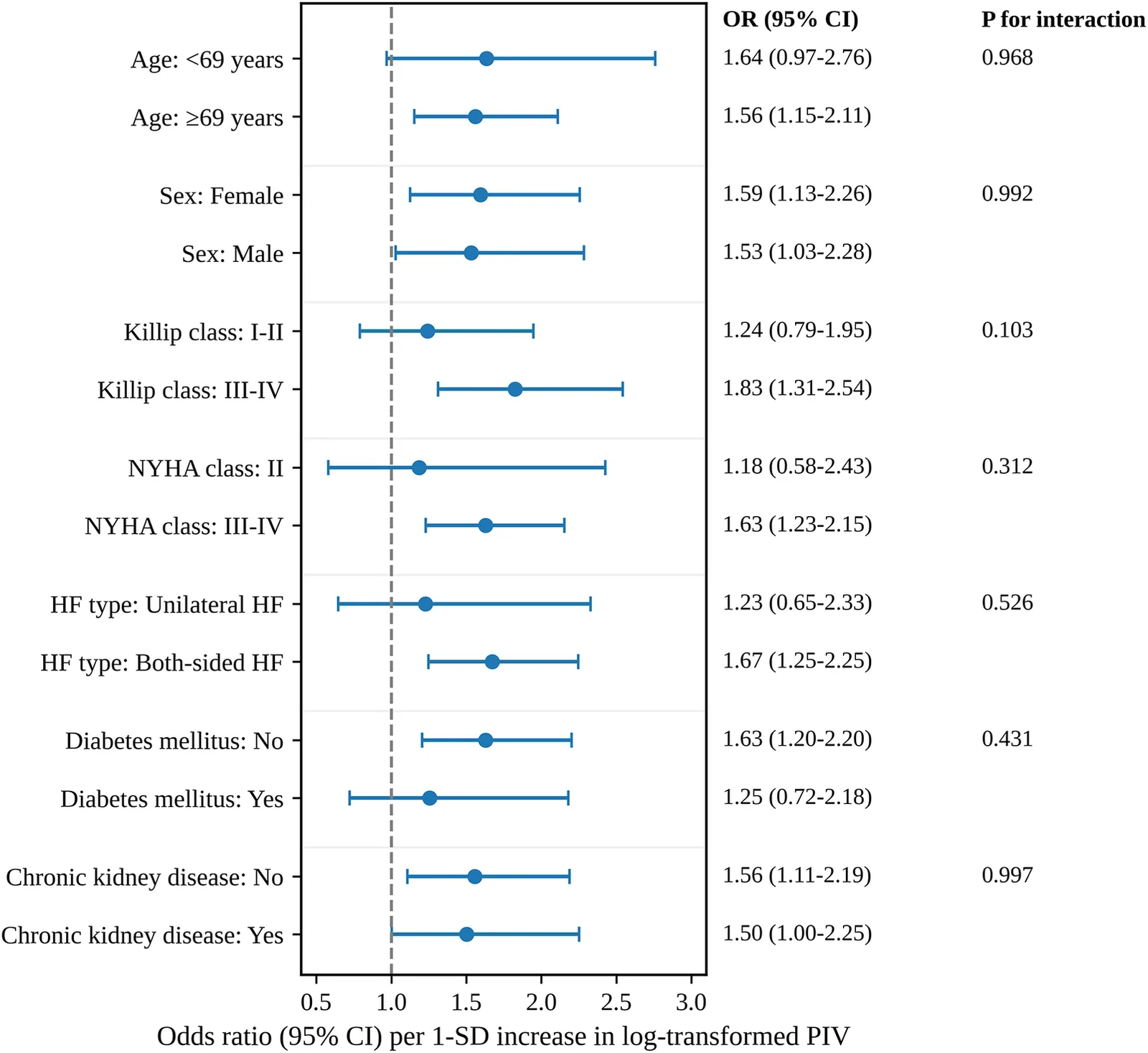
Subgroup analysis of the association between PIV and 6-month all-cause mortality in the derivation cohort. Forest plots show odds ratios and 95% confidence intervals for 6-month mortality per 1-standard-deviation increase in log-transformed PIV across subgroups defined by age, sex, Killip class, New York Heart Association class, heart failure type, diabetes mellitus, and chronic kidney disease. The vertical dashed line indicates an odds ratio of 1. *P* values for interaction are displayed for each subgroup comparison. PIV, pan-immune-inflammation value; HF, heart failure; NYHA, New York Heart Association.

The association between PIV and mortality was generally consistent across subgroups defined by age, sex, Killip class, NYHA class, HF type, DM, and CKD (Supplementary Table S2 and Figure 5). All interaction *P* > 0.05, indicating no clear effect modification. Sensitivity analyses using SIRI and SII produced results consistent with the primary analysis (Supplementary Table S3). In fully adjusted models, each 1-SD increase in log-transformed SIRI and SII was associated with higher mortality, with ORs of 1.47 (95% CI, 1.13–1.91; *P* = 0.004) and 1.54 (95% CI, 1.17–2.01; *P* = 0.002), respectively. The highest quartiles of SIRI and SII were also associated with increased mortality (OR, 7.93 and 3.90, respectively).

PIV remained associated with both 28-day mortality (OR, 1.85; 95% CI, 1.32–2.61) and 3-month mortality (OR, 1.79; 95% CI, 1.30–2.47) in the fully adjusted models (Supplementary Table S4). PIV was strongly correlated with SIRI and SII (Spearman r = 0.908 and 0.884, respectively), supporting their evaluation in separate models. The variance inflation factor for log-transformed PIV was 1.04, indicating no meaningful multicollinearity (Supplementary Table S5). In the Firth penalized logistic regression sensitivity analysis, higher PIV remained independently associated with 6-month mortality (OR per 1-SD increase in log-transformed PIV, 1.55; profile penalized-likelihood 95% CI, 1.19–2.01; penalized-likelihood ratio *P* = 0.001), closely corresponding to the conventional maximum-likelihood estimate (Supplementary Methods S2).

### External validation in MIMIC-IV

**Figure 6:**
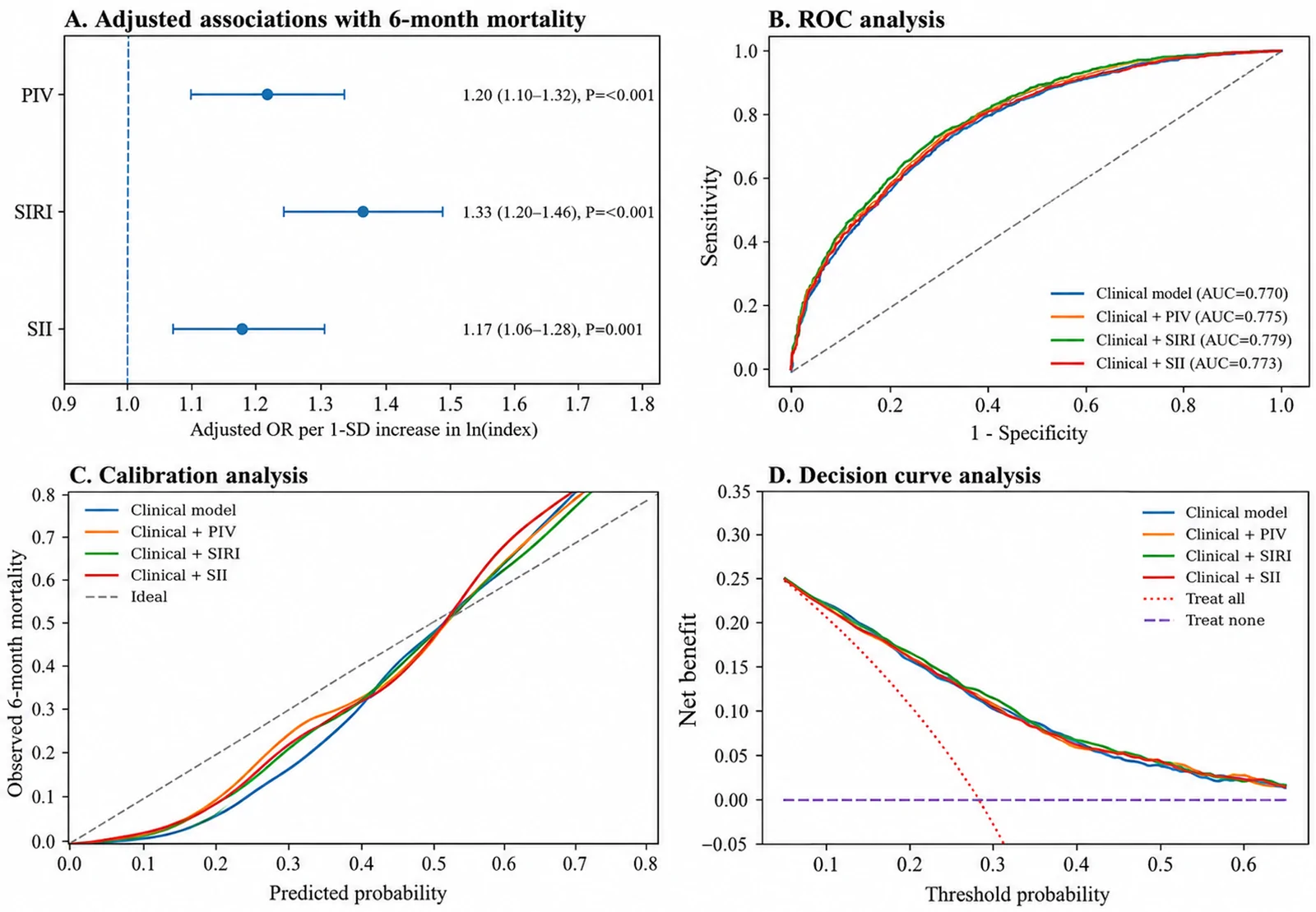
External validation of immune-inflammatory indices in the MIMIC-IV cohort. **(A)** Fully adjusted associations between immune-inflammatory indices and 6-month all-cause mortality in the MIMIC-IV external validation cohort, expressed as odds ratios (ORs) with 95% confidence intervals (CIs) per 1-standard deviation (SD) increase in ln-transformed PIV, SIRI, and SII. **(B)** Receiver operating characteristic (ROC) curves comparing discrimination of the clinical model alone and the clinical model augmented with PIV, SIRI, or SII for predicting 6-month all-cause mortality. The area under the curve (AUC) was 0.770 for the clinical model, 0.775 for the clinical model plus PIV, 0.779 for the clinical model plus SIRI, and 0.773 for the clinical model plus SII. **(C)** Calibration curves of the clinical model alone and the three index-augmented models for 6-month all-cause mortality. The dashed diagonal line indicates ideal agreement between predicted and observed risk. **(D)** Decision curve analysis (DCA) of the clinical model and the three index-augmented models. All augmented models provided slightly improved net benefit over the clinical model alone across a range of threshold probabilities. The red dotted line represents the treat-all strategy, and the purple dashed line represents the treat-none strategy. PIV, pan-immune-inflammation value; SIRI, systemic inflammation response index; SII, systemic immune-inflammation index; ROC, receiver operating characteristic; AUC, area under the curve; DCA.

Among 9,703 adults with a first AMI hospitalization in MIMIC-IV, 2,679 patients had the laboratory variables required to calculate the immune-inflammatory indices and constituted the complete-case validation cohort; 773 died within 6 months (Figure 1). The MIMIC-IV cohort had a substantially higher 6-month mortality rate than the derivation cohort and represented a different clinical case mix. Moreover, because several derivation-cohort predictors and a directly comparable definition of conservative treatment were unavailable, the MIMIC-IV-specific clinical model was not identical to the derivation-cohort model. Accordingly, the external analysis evaluated whether the associations and incremental predictive patterns were reproducible in an independent setting rather than assessing the transportability of unchanged model coefficients. Because several derivation-cohort variables were not structurally available in MIMIC-IV, external validation used a cohort-specific adjustment model based on available demographic, admission-related, and comorbidity variables. Non-survivors were older, more frequently admitted to the intensive care unit, and had a higher Charlson comorbidity index (Supplementary Table S6). Median PIV was 1,239.8 in non-survivors vs. 720.7 in survivors, while SIRI and SII were also significantly higher (all *P* < 0.001). External validation results are presented in Table 4 and Figure 6. In the MIMIC-IV external validation cohort, all three immune-inflammatory indices remained independently associated with 6-month all-cause mortality after multivariable adjustment, with adjusted ORs of 1.20 (95% CI, 1.10–1.32) for PIV, 1.33 (95% CI, 1.20–1.46) for SIRI, and 1.17 (95% CI, 1.06–1.28) for SII (Figure 6A; Table 4). In discrimination analyses, the clinical model alone yielded an AUROC of 0.770, which increased to 0.775 after adding PIV, 0.779 after adding SIRI, and 0.773 after adding SII (Figure 6B; Table 4). Calibration analyses showed acceptable agreement between predicted and observed risks across models, and DCA indicated modest net-benefit improvement after incorporation of the immune-inflammatory indices (Figures 6C,D). The association of PIV remained significant for 28-day, 3-month, 6-month, and 1-year mortality, with fully adjusted ORs ranging from 1.14 to 1.21 (Supplementary Table S7). SIRI and SII showed similar temporal consistency. Across the derivation and MIMIC-IV cohorts, the differences in predictive performance among the PIV-, SIRI-, and SII-augmented models were small. PIV did not consistently outperform SIRI or SII across discrimination and incremental performance measures; therefore, these comparisons should be regarded as supportive analyses rather than evidence of the superiority of PIV.

**Table 4:** External validation of immune-inflammatory indices in the MIMIC-IV cohort. Part A Preliminary associations with 6-month all-cause mortality.

Index	Survivors	Non-survivors	*P* value	AUROC (95% CI)
PIV	720.7 (325.4, 1709.4)	1239.8 (487.0, 3021.2)	<0.001	0.600 (0.576–0.626)
SIRI	3.36 (1.62, 7.79)	6.62 (2.85, 14.94)	<0.001	0.635 (0.612–0.659)
SII	1296.6 (680.0, 2547.3)	1961.4 (968.6, 3984.9)	<0.001	0.597 (0.571–0.621)

Part B Multivariable logistic regression models for 6-month all-cause mortality						
Index	Model	n	events	OR (95% CI)	*P* value	AIC
PIV	Model 1	2679	773	1.33 (1.21–1.45)	<0.001	3183.0
PIV	Model 2	2679	773	1.23 (1.12–1.35)	<0.001	2991.0
PIV	Model 3	2679	773	1.20 (1.10–1.32)	<0.001	2732.3
SIRI	Model 1	2679	773	1.56 (1.42–1.71)	<0.001	3127.6
SIRI	Model 2	2679	773	1.43 (1.30–1.57)	<0.001	2954.9
SIRI	Model 3	2679	773	1.33 (1.20–1.46)	<0.001	2713.6
SII	Model 1	2679	773	1.32 (1.21–1.44)	<0.001	3183.8
SII	Model 2	2679	773	1.21 (1.10–1.32)	<0.001	2994.5
SII	Model 3	2679	773	1.17 (1.06–1.28)	0.001	2737.1

Part C Quartile analyses using fully adjusted Model 3						
Index	Quartile	n	events	OR (95% CI)	*P* value	*P* for trend
PIV	Q2 vs. Q1	2679	773	1.17 (0.88–1.55)	0.289	<0.001
PIV	Q3 vs. Q1	2679	773	1.26 (0.96–1.67)	0.101	<0.001
PIV	Q4 vs. Q1	2679	773	1.89 (1.44–2.48)	<0.001	<0.001
SIRI	Q2 vs. Q1	2679	773	1.18 (0.88–1.59)	0.277	<0.001
SIRI	Q3 vs. Q1	2679	773	1.64 (1.23–2.18)	<0.001	<0.001
SIRI	Q4 vs. Q1	2679	773	2.22 (1.68–2.95)	<0.001	<0.001
SII	Q2 vs. Q1	2679	773	0.99 (0.74–1.31)	0.938	<0.001
SII	Q3 vs. Q1	2679	773	1.09 (0.83–1.44)	0.533	<0.001
SII	Q4 vs. Q1	2679	773	1.62 (1.24–2.13)	<0.001	<0.001

Part D Incremental predictive value compared with the clinical model									
Model	AUROC (95% CI)	*Δ*AUROC	*P* for *Δ*AUROC	Brier score	AIC	NRI (95% CI)	*P* for NRI	IDI (95% CI)	*P* for IDI
Clinical model	0.770 (0.753–0.788)			0.167	2785.9	Reference		Reference	
Clinical model + PIV	0.775 (0.755–0.792)	0.004	0.028	0.166	2771.3	0.244 (0.159–0.320)	<0.001	0.005 (0.002–0.008)	0.006
Clinical model + SIRI	0.779 (0.762–0.798)	0.009	0.006	0.165	2753.0	0.337 (0.255–0.422)	<0.001	0.011 (0.006–0.016)	<0.001
Clinical model + SII	0.773 (0.756–0.792)	0.003	0.072	0.166	2776.5	0.263 (0.172–0.345)	<0.001	0.004 (0.001–0.006)	0.018

ORs in Part B are shown per 1-SD increase in natural log-transformed PIV, SIRI, or SII. Model 1 was unadjusted. Model 2 was adjusted for age, sex, race, admission type, and ICU admission. Model 3 was further adjusted for insurance, Charlson comorbidity index, chronic heart failure, diabetes mellitus, chronic kidney disease, acute renal failure, and history of myocardial infarction. Continuous NRI and IDI were calculated relative to the MIMIC-IV-specific clinical model.

AIC, Akaike information criterion; AUROC, area under the receiver operating characteristic curve; CI, confidence interval; ICU, intensive care unit; IDI, integrated discrimination improvement; IQR, interquartile range; MIMIC-IV, Medical Information Mart for Intensive Care IV; NRI, net reclassification improvement; OR, odds ratio; PIV, pan-immune-inflammation value; SD, standard deviation; SII, systemic immune-inflammation index; SIRI, systemic inflammation response index.

## Discussion

In this two-cohort study, higher PIV was consistently associated with 6-month all-cause mortality after AMI. The association persisted after adjustment for conventional clinical predictors and was directionally supported by SIRI/SII analyses and MIMIC-IV validation. These findings are consistent with evidence that residual inflammatory activity and circulating leukocyte-derived indices may capture prognostic information beyond traditional cardiovascular risk factors (15, 16). However, the observed association does not establish a pathogenic role for PIV; rather, PIV may serve as a peripheral surrogate marker of the systemic immune-inflammatory and thrombotic status associated with adverse outcomes after AMI (17, 18).

Previous AMI risk-stratification studies have mainly relied on demographic characteristics, hemodynamic status, cardiac dysfunction, renal impairment, and comorbidity burden. Khera et al. developed machine-learning models for post-AMI mortality using routinely available clinical variables (19), while Tomasdottir et al. identified age and renal dysfunction as important mortality markers in acute coronary syndrome (ACS) patients using electronic health record data (20). Song et al. similarly showed that cardiac functional grade, acute renal failure, and diabetes-related complications were strongly associated with in-hospital mortality after ST-segment elevation myocardial infarction (STEMI) (21). Compared with these studies (22), the present work specifically evaluated whether an immune-inflammatory index could provide additional prognostic information beyond a prespecified clinical model.

After AMI, ischemic cardiomyocyte necrosis triggers a sterile inflammatory cascade characterized by the release of damage-associated molecular patterns, activation of innate immune signaling, and recruitment of circulating immune cells to the injured myocardium. At the molecular level, damage-associated molecular patterns released from necrotic cardiomyocytes may activate pattern-recognition receptor pathways, including Toll-like receptor-related signaling, nuclear factor-*κ*B activation, interferon-related innate immune responses, and inflammasome-associated cytokine release (23, 24). These pathways may promote the production of interleukin-1β, interleukin-6, tumor necrosis factor-α, and other inflammatory mediators, thereby amplifying leukocyte recruitment, endothelial activation, and systemic inflammatory responses after AMI (8, 9). In parallel, platelet-derived mediators, including P-selectin and CD40 ligand, may strengthen platelet–leukocyte interactions and link vascular inflammation with thrombosis (25). Neutrophils are among the earliest responders and may aggravate myocardial injury through oxidative stress, protease release, neutrophil extracellular trap formation, endothelial injury, and microvascular obstruction; however, those factors also participate in the initiation of tissue clearance and subsequent repair (26, 27). Monocytes are then recruited and differentiate into macrophage subsets that regulate phagocytosis of necrotic debris, inflammatory resolution, fibroblast activation, and extracellular matrix remodeling. An excessive or prolonged monocyte/macrophage response may contribute to adverse ventricular remodeling and heart failure progression (28). Platelets are not only mediators of coronary thrombosis but also active immune modulators that interact with leukocytes, amplify vascular inflammation, promote endothelial activation, and facilitate immunothrombotic responses after plaque rupture (25). In contrast, lymphocyte reduction may reflect stress-induced immune dysregulation and impaired adaptive immune control. Accordingly, an index combining neutrophils, monocytes, platelets, and lymphocytes may provide a composite representation of the circulating immune-inflammatory and thrombotic response after AMI. Nevertheless, PIV is a calculated marker and should not be interpreted as a direct mediator of myocardial injury, thrombosis, or ventricular remodeling. Previous studies have similarly shown associations of SII and SIRI with adverse cardiovascular outcomes (29, 30). Compared with these indices, PIV simultaneously incorporates four immune-thrombotic components, which may provide a broader representation of the systemic immune-inflammatory burden in AMI. Chronic glycemic exposure may provide an additional metabolic context for immune-inflammatory risk after AMI. Recent HbA1c-based categorical and continuous analyses suggest that metabolic and microvascular vulnerability may increase across the glycemic spectrum, including within the prediabetic range (31, 32). Potential links include oxidative stress, endothelial dysfunction, low-grade inflammation, and impaired microvascular integrity, although these mechanisms were not directly demonstrated in the cited studies (31, 32). HbA1c and PIV may therefore reflect complementary risk dimensions: longer-term glycemic exposure and the circulating immune-inflammatory and thrombotic state around the time of AMI, respectively. However, current evidence does not establish a direct effect of HbA1c on myocardial inflammation. Prospective AMI studies should evaluate the independent, interactive, and incremental prognostic value of HbA1c and PIV.

Blood-cell-derived inflammatory indices such as NLR and the platelet-to-lymphocyte ratio have previously been associated with adverse outcomes in acute coronary syndrome (33, 34). while SII and SIRI have been proposed as markers of inflammatory burden in coronary artery disease and acute coronary syndrome (35). In the present study, SIRI and SII were evaluated as complementary indices, and their predictive performance was generally comparable with that of PIV. PIV integrates neutrophils, monocytes, platelets, and lymphocytes within a single metric and has shown prognostic value in inflammatory and immune-related clinical settings (36, 37), whereas SII does not include monocytes and SIRI does not include platelets. This broader cellular composition provides a rationale for evaluating PIV as a composite marker of circulating immune-inflammatory and thrombotic status; however, it should not be interpreted as evidence that PIV has superior predictive performance or clinical utility. Because a direct comparison with NLR was not performed in the present study, appropriately designed head-to-head studies are required to determine whether PIV provides meaningful prognostic advantages over established inflammatory indices.

Consistent with this broader immune-thrombotic interpretation, the clinical value of PIV lies not in replacing established AMI risk factors, but in complementing them. A strength of the present study is that the reference clinical model was prespecified according to clinical relevance, routine availability, and established AMI prognostic relevance, rather than derived from outcome-driven variable selection. This design allowed the incremental value of PIV, SIRI, and SII to be evaluated against a conventional clinical comparator. Although the MIMIC-IV model required adaptation because several derivation-cohort variables were not available as comparable structured fields, both cohorts followed the same modeling principle (38, 39). In the derivation cohort, adding PIV to the reference clinical model increased the AUROC from 0.844 to 0.854, although this difference was not statistically significant. Bootstrap internal validation yielded optimism-corrected AUROCs of 0.813 and 0.823, respectively, with a similarly small corrected increment of 0.0106. Continuous NRI and IDI were exploratory and should be interpreted alongside the small and nonsignificant AUROC change. Improvements in model fit, reclassification indices, and decision-curve estimates suggest that PIV may provide additional prognostic information not fully captured by conventional clinical variables; however, the magnitude of this improvement was modest. Therefore, PIV should not be considered a replacement for established clinical risk factors or validated risk scores. Its potential value may instead lie in serving as a readily available complementary marker when interpreted together with demographic characteristics, cardiac functional status, comorbidity burden, and conventional laboratory findings. The persistent association between PIV and mortality in MIMIC-IV supports the transportability of the association, but it does not establish that incorporating PIV into routine clinical assessment improves treatment decisions or patient outcomes. Prospective studies should determine whether PIV-guided risk stratification provides clinically meaningful benefit beyond existing approaches (40, 41).

### Limitations and future directions

Several limitations should be acknowledged. First, there are limitations inherent to the retrospective observational design. These studies are at risk of selection bias and cannot be used to infer causality. Selection of the study population was potentially affected by restricting the derivation cohort to conservatively treated patients, inclusion based on admission blood-cell measurements and follow-up availability, and complete-case selection in both cohorts. Thus, the studied patients may not be entirely representative of the broader AMI population. While multivariable adjustment, sensitivity analyses, and external validation were used to reduce bias from confounding, residual and unmeasured confounding cannot be ruled out. These may include medication use and/or adherence, differences in treatment intensity and reperfusion-related characteristics, concomitant infection/inflammatory disease, nutritional status and frailty, and differences in post-discharge management. For these reasons, the PIV-mortality association should be considered prognostic, rather than causal. Second, the single-center derivation cohort was restricted to conservatively treated patients with AMI. Its case mix, treatment strategy, and relatively low event rate may differ from those of contemporary populations receiving routine reperfusion therapy; therefore, the performance of the derivation model should not be directly extrapolated to other AMI populations or treatment pathways. Third, although MIMIC-IV provided an independent external validation cohort, it represented a broader real-world AMI population rather than an exact replication of the derivation cohort. Reperfusion status could not be defined using a validated algorithm comparable with that used in the derivation cohort, and several important variables, including Killip class, NYHA class, symptom-to-door time, and HF type, were unavailable as directly comparable structured fields. Consequently, the external analysis required a cohort-specific adjustment model. These differences indicate that the MIMIC-IV findings support the reproducibility of the prognostic associations, but do not constitute direct validation of an unchanged prediction model. Because patient characteristics, treatment strategies, blood-sampling practices, and baseline mortality risks may vary across settings, local validation and, where necessary, recalibration would be required before PIV is incorporated into clinical risk-assessment pathways. Fourth, PIV, SIRI, and SII were calculated from blood-cell measurements obtained around admission, and dynamic changes during hospitalization were not evaluated. A single baseline measurement may not fully capture the evolving immune-inflammatory response after AMI. Fifth, only 57 deaths occurred relative to the number of model parameters, raising concerns about overfitting and imprecision. Although bootstrap validation indicated some optimism and Firth regression yielded a similar PIV estimate, the limited event count remains a source of uncertainty, and the NRI and IDI findings should be considered exploratory. Finally, PIV is a composite index derived from peripheral blood-cell counts and does not directly measure immune-cell phenotypes, cytokine activity, tissue-level inflammation, or causal pathways. The biological roles of its individual components should therefore not be interpreted as evidence that PIV itself has a direct pathogenic effect. Prospective studies incorporating serial measurements, immune-cell phenotyping, cytokine profiling, and relevant treatment information are needed to determine whether PIV primarily serves as a prognostic surrogate or reflects specific biological pathways associated with adverse outcomes after AMI. PIV was prespecified as the primary index, while SIRI and SII were evaluated as complementary indices. Although NLR is a widely used inflammatory marker, it was not included in the prespecified comparative analyses. Consequently, the present study cannot determine whether PIV provides greater predictive value than NLR or other established blood-cell-derived indices. Future studies should prospectively compare these indices using a uniform analytical framework and evaluate whether any observed differences are sufficiently large to influence clinical risk stratification.

## Conclusion

In this retrospective study, higher PIV was independently associated with 6-month all-cause mortality after AMI, with a consistent association observed in MIMIC-IV. The incremental predictive improvement beyond conventional clinical factors was modest and remained small after bootstrap correction, and PIV should therefore be regarded as a potential complementary marker rather than a stand-alone risk-stratification tool. These findings demonstrate a prognostic association but do not establish causality or support immediate use of PIV in clinical decision-making. Prospective multicenter studies are required to determine whether incorporating PIV into established risk-assessment strategies provides clinically meaningful benefit.

## Data Availability

The raw data supporting the conclusions of this article will be made available by the authors, without undue reservation.
