# Supplementary material for: Incremental prognostic value of pan-immune-inflammation value in conservatively treated acute myocardial infarction: derivation and MIMIC-IV validation
Source: Front Cardiovasc Med. 2026 Sep 10;13:1935766. doi: 10.3389/fcvm.2026.1935766 (PMC13602858; doi:10.3389/fcvm.2026.1935766)
Supplement: Supplementary file 1 [file Supplementaryfile1.docx]

**Supplementary Methods S1. Construction of the Reference Clinical Models**

The reference clinical models were constructed a priori to evaluate whether immune-inflammatory indices provided incremental prognostic information beyond conventional clinical predictors. Candidate variables were selected according to clinical relevance, routine availability at or near hospital admission, and established prognostic associations with mortality after acute myocardial infarction (AMI), as supported by established ACS risk stratification systems and comorbidity measures (1-8). No data-driven variable selection procedure was used to define the reference clinical models.

In the derivation cohort, the reference clinical model included age category, sex, Killip class, New York Heart Association (NYHA) class, Charlson comorbidity index, previous myocardial infarction, chronic heart failure, diabetes mellitus, chronic kidney disease, and acute renal failure. These variables were chosen to represent demographic risk, cardiac functional status, and comorbidity burden. The selection was consistent with prior evidence that age, hemodynamic or heart-failure severity, cardiovascular history, diabetes, renal dysfunction, and comorbidity burden are important predictors of mortality after AMI or acute coronary syndrome (1-10).

The MIMIC-IV external validation model followed the same construction principle but was adapted according to structured variable availability. Several derivation-cohort variables, including Killip class, NYHA class, symptom-to-door time, and heart failure type, were not available as directly comparable structured variables in MIMIC-IV. Therefore, the MIMIC-IV reference clinical model included age, sex, race, admission type, intensive care unit admission, insurance, Charlson comorbidity index, chronic heart failure, diabetes mellitus, chronic kidney disease, acute renal failure, and history of myocardial infarction. These variables were selected to capture demographic characteristics, admission severity, socioeconomic proxy information, and comorbidity burden using reproducible structured data (8,11,12).

For incremental prediction analyses, PIV, SIRI, and SII were added separately to the corresponding reference clinical model in each cohort. Model performance was compared between the reference clinical model and each index-extended model using discrimination, calibration, clinical utility, and reclassification metrics. This design allowed the incremental value of immune-inflammatory indices to be evaluated under a consistent modeling principle while accounting for cohort-specific differences in variable availability.

**Supplementary methods Figure S1**

**Supplementary methods Figure S1 Conceptual workflow for construction and application of the reference clinical models. AMI, acute myocardial infarction; PIV, pan-immune-inflammation value; SII, systemic immune-inflammation index; SIRI, systemic inflammation response index.**

**Supplementary Methods Table S1 Construction principles and covariate availability of the reference clinical models**

| **Model domain** | **Derivation cohort** | **MIMIC-IV cohort** | **Reason for inclusion or adaptation** |
| --- | --- | --- | --- |
| Demographic risk | Age category; sex | Age; sex; race | Age and sex are core baseline prognostic factors in AMI/ACS risk models. Race was included only in MIMIC-IV because it was available as a structured demographic variable [2-6,12]. |
| Cardiac or admission severity | Killip class; NYHA class | Admission type; ICU admission | Killip/heart-failure severity is a recognized mortality-related domain after AMI. In MIMIC-IV, admission type and ICU admission were used as reproducible structured proxies for acute illness severity because Killip and NYHA classes were not directly available [1,2,4-6,12]. |
| Comorbidity burden | Charlson comorbidity index | Charlson comorbidity index | The Charlson comorbidity index summarizes chronic disease burden and is widely used for prognostic adjustment in longitudinal and administrative data analyses [7,8]. |
| Cardiovascular history | Previous myocardial infarction; chronic heart failure | History of myocardial infarction; chronic heart failure | Prior myocardial infarction and chronic heart failure reflect established cardiovascular disease burden and have prognostic relevance in AMI/ACS populations [3-6]. |
| Metabolic and renal comorbidities | Diabetes mellitus; chronic kidney disease; acute renal failure | Diabetes mellitus; chronic kidney disease; acute renal failure | Diabetes and renal dysfunction are common comorbidities associated with adverse outcomes after AMI/ACS and were available in both datasets [6,9,10]. |
| Variables not directly harmonized | Symptom-to-door time; heart failure type | Not directly comparable in structured MIMIC-IV tables | These variables were available in the derivation cohort but were not reproducibly extractable as directly comparable structured variables in MIMIC-IV. The external validation model therefore used MIMIC-IV-available covariates under the same modeling principle [11,12]. |

**Note: The clinical models were prespecified to provide a conventional reference for evaluating the incremental value of PIV, SIRI, and SII. The two cohorts followed the same modeling principle, but the MIMIC-IV model was adapted to structured variable availability.**

**References**

1. Killip T 3rd, Kimball JT. Treatment of myocardial infarction in a coronary care unit: a two-year experience with 250 patients. Am J Cardiol. 1967;20(4):457-464. doi:10.1016/0002-9149(67)90023-9. PMID: 6059183.

2. Morrow DA, Antman EM, Charlesworth A, Cairns R, Murphy SA, de Lemos JA, et al. TIMI risk score for ST-elevation myocardial infarction: a convenient, bedside, clinical score for risk assessment at presentation: an InTIME II trial substudy. Circulation. 2000;102(17):2031-2037. doi:10.1161/01.CIR.102.17.2031. PMID: 11044416.

3. Antman EM, Cohen M, Bernink PJ, McCabe CH, Horacek T, Papuchis G, et al. The TIMI risk score for unstable angina/non-ST elevation MI: a method for prognostication and therapeutic decision making. JAMA. 2000;284(7):835-842. doi:10.1001/jama.284.7.835. PMID: 10938172.

4. Granger CB, Goldberg RJ, Dabbous O, Pieper KS, Eagle KA, Cannon CP, et al. Predictors of hospital mortality in the Global Registry of Acute Coronary Events. Arch Intern Med. 2003;163(19):2345-2353. doi:10.1001/archinte.163.19.2345. PMID: 14581255.

5. Eagle KA, Lim MJ, Dabbous OH, Pieper KS, Goldberg RJ, Van de Werf F, et al. A validated prediction model for all forms of acute coronary syndrome: estimating the risk of 6-month postdischarge death in an international registry. JAMA. 2004;291(22):2727-2733. doi:10.1001/jama.291.22.2727. PMID: 15187054.

6. Fox KAA, Dabbous OH, Goldberg RJ, Pieper KS, Eagle KA, Van de Werf F, et al. Prediction of risk of death and myocardial infarction in the six months after presentation with acute coronary syndrome: prospective multinational observational study (GRACE). BMJ. 2006;333(7578):1091. doi:10.1136/bmj.38985.646481.55. PMID: 17032691.

7. Charlson ME, Pompei P, Ales KL, MacKenzie CR. A new method of classifying prognostic comorbidity in longitudinal studies: development and validation. J Chronic Dis. 1987;40(5):373-383. doi:10.1016/0021-9681(87)90171-8. PMID: 3558716.

8. Quan H, Sundararajan V, Halfon P, Fong A, Burnand B, Luthi JC, et al. Coding algorithms for defining comorbidities in ICD-9-CM and ICD-10 administrative data. Med Care. 2005;43(11):1130-1139. doi:10.1097/01.mlr.0000182534.19832.83. PMID: 16224307.

9. Franklin K, Goldberg RJ, Spencer F, Klein W, Budaj A, Brieger D, et al. Implications of diabetes in patients with acute coronary syndromes. The Global Registry of Acute Coronary Events. Arch Intern Med. 2004;164(13):1457-1463. doi:10.1001/archinte.164.13.1457. PMID: 15249353.

10. Anavekar NS, McMurray JJV, Velazquez EJ, Solomon SD, Kober L, Rouleau JL, et al. Relation between renal dysfunction and cardiovascular outcomes after myocardial infarction. N Engl J Med. 2004;351(13):1285-1295. doi:10.1056/NEJMoa041365. PMID: 15385655.

11. Johnson AEW, Pollard TJ, Shen L, Li-wei HL, Feng M, Ghassemi M, et al. MIMIC-III, a freely accessible critical care database. Sci Data. 2016;3:160035. doi:10.1038/sdata.2016.35. PMID: 27219127.

12. Johnson AEW, Bulgarelli L, Shen L, Gayles A, Shammout A, Horng S, et al. MIMIC-IV, a freely accessible electronic health record dataset. Sci Data. 2023;10(1):1. doi:10.1038/s41597-022-01899-x. PMID: 36596836.
