# Supplementary material for: Incremental prognostic value of pan-immune-inflammation value in conservatively treated acute myocardial infarction: derivation and MIMIC-IV validation
Source: Front Cardiovasc Med. 2026 Sep 10;13:1935766. doi: 10.3389/fcvm.2026.1935766 (PMC13602858; doi:10.3389/fcvm.2026.1935766)
Supplement: Supplementary file 2 [file Supplementaryfile2.docx]

**Supplementary Methods S2. Bootstrap internal validation and penalized regression sensitivity analysis**

To quantify potential overfitting in the derivation cohort, internal validation was performed using 1,000 nonparametric bootstrap resamples drawn with replacement. In every resample, the prespecified reference clinical model and the model augmented with pan-immune-inflammation value (PIV) were refitted using the same predictor definitions and transformations as in the primary analysis. Model performance was calculated in both the bootstrap resample and the original derivation cohort. Optimism was defined as the difference between bootstrap-sample and original-cohort performance, averaged across the 1,000 resamples, and subtracted from the apparent estimate. Optimism-corrected area under the receiver operating characteristic curve (AUROC), Brier score, calibration slope, and calibration intercept were calculated. Identical paired resamples were used for the clinical and PIV-augmented models.

Because only 57 deaths occurred in the derivation cohort, Firth penalized logistic regression was additionally performed as a small-sample sensitivity analysis for the fully adjusted continuous-PIV association. The model included age category, sex, Killip class, New York Heart Association class, Charlson comorbidity index, symptom-to-door time, both-sided heart failure, previous myocardial infarction, chronic heart failure, diabetes mellitus, chronic kidney disease, and standardized natural log-transformed PIV. Profile penalized-likelihood 95% confidence intervals were reported. Exact event dates and individual censoring times were unavailable in the derivation dataset; therefore, Cox proportional hazards regression could not be validly estimated, and the analyses remained based on mortality defined at prespecified fixed follow-up horizons.

**Supplementary Methods Table S2 Bootstrap internal validation of the primary PIV model comparison**

| **Model** | **Apparent AUROC** | **Optimism-corrected AUROC** | **Apparent Brier score** | **Optimism-corrected Brier score** | **Optimism-corrected calibration slope** | **Optimism-corrected calibration intercept** |
| --- | --- | --- | --- | --- | --- | --- |
| Reference clinical model | 0.844 | 0.813 | 0.0247 | 0.0259 | 0.829 | 0.010 |
| Reference clinical model + PIV | 0.854 | 0.823 | 0.0241 | 0.0255 | 0.831 | 0.008 |

**Notes:** Internal validation used 1,000 paired bootstrap resamples. The apparent AUROC increment after adding PIV was 0.010; the optimism-corrected increment was 0.0106. Apparent calibration slopes and intercepts were 1 and 0 by construction; only optimism-corrected values are shown. Calibration intercepts are expressed on the log-odds scale.

**Abbreviations:** AUROC, area under the receiver operating characteristic curve; PIV, pan-immune-inflammation value.

**Supplementary Methods Table S3 Firth penalized logistic regression sensitivity analysis of the PIV-mortality association**

| **Analytical method** | **OR per 1-SD increase in log-transformed PIV** | **95% CI** | **P value** |
| --- | --- | --- | --- |
| Conventional maximum-likelihood logistic regression | 1.56 | 1.19-2.04 | 0.001 |
| Firth penalized logistic regression | 1.55 | 1.19-2.01 | 0.001 |

**Notes:** Both estimates were obtained from the same fully adjusted continuous-PIV model. The conventional estimate is based on maximum-likelihood logistic regression. The Firth estimate uses bias-reduced penalized likelihood, a profile penalized-likelihood 95% confidence interval, and a penalized-likelihood ratio P value.

**Abbreviations:** CI, confidence interval; OR, odds ratio; PIV, pan-immune-inflammation value; SD, standard deviation.
