# Supplementary material for: Incremental prognostic value of pan-immune-inflammation value in conservatively treated acute myocardial infarction: derivation and MIMIC-IV validation
Source: Front Cardiovasc Med. 2026 Sep 10;13:1935766. doi: 10.3389/fcvm.2026.1935766 (PMC13602858; doi:10.3389/fcvm.2026.1935766)
Supplement: Supplementary file 3 [file Table1.docx]

**Supplementary Table S1 Baseline characteristics according to 6-month all-cause mortality**

| **Characteristic** | **Overall (n = 2008)** | **Survivors (n = 1951)** | **Non-survivors (n = 57)** | **P value** |
| --- | --- | --- | --- | --- |
| ***Demographic and admission characteristics*** |  |  |  |  |
| Age group, years |  |  |  | 0.689 |
| *21-29* | *4 (0.2)* | *4 (0.2)* | *0 (0.0)* |  |
| *29-39* | *12 (0.6)* | *12 (0.6)* | *0 (0.0)* |  |
| *39-49* | *56 (2.8)* | *55 (2.8)* | *1 (1.8)* |  |
| *49-59* | *106 (5.3)* | *102 (5.2)* | *4 (7.0)* |  |
| *59-69* | *368 (18.3)* | *358 (18.3)* | *10 (17.5)* |  |
| *69-79* | *715 (35.6)* | *696 (35.7)* | *19 (33.3)* |  |
| *79-89* | *646 (32.2)* | *626 (32.1)* | *20 (35.1)* |  |
| *89-110* | *101 (5.0)* | *98 (5.0)* | *3 (5.3)* |  |
| Sex |  |  |  | 0.172 |
| *Female* | *1163 (57.9)* | *1135 (58.2)* | *28 (49.1)* |  |
| *Male* | *845 (42.1)* | *816 (41.8)* | *29 (50.9)* |  |
| Symptom-to-door time category |  |  |  | 0.924 |
| *Category 1* | *1860 (92.6)* | *1807 (92.6)* | *53 (93.0)* |  |
| *Category 2* | *120 (6.0)* | *117 (6.0)* | *3 (5.3)* |  |
| *Category 3* | *20 (1.0)* | *19 (1.0)* | *1 (1.8)* |  |
| *Category 4* | *6 (0.3)* | *6 (0.3)* | *0 (0.0)* |  |
| *Category 5* | *2 (0.1)* | *2 (0.1)* | *0 (0.0)* |  |
| Temperature, deg C | 36.3 (36.2, 36.5) | 36.3 (36.2, 36.5) | 36.3 (36.0, 36.5) | 0.363 |
| Heart rate, beats/min | 82 (70, 98) | 82 (70, 98) | 82 (72, 100) | 0.450 |
| Respiratory rate, breaths/min | 19 (18, 19) | 19 (18, 19) | 19 (18, 20) | 0.062 |
| Systolic blood pressure, mmHg | 130 (113, 146) | 130 (113, 147) | 126 (110, 142) | 0.286 |
| Diastolic blood pressure, mmHg | 76 (65, 85) | 76 (66, 85) | 73 (62, 84) | 0.145 |
| Mean arterial pressure, mmHg | 93.3 (83.3, 104.7) | 93.3 (83.3, 104.7) | 90.0 (78.7, 101.3) | 0.140 |
| Weight, kg | 50.0 (45.0, 60.0) | 50.0 (45.0, 60.0) | 50.0 (45.0, 60.0) | 0.892 |
| Height, m | 1.56 (1.50, 1.62) | 1.56 (1.50, 1.62) | 1.58 (1.53, 1.63) | 0.596 |
| BMI, kg/m2 | 20.76 (18.49, 23.44) | 20.76 (18.49, 23.44) | 20.72 (18.49, 23.14) | 0.920 |
| ***Heart failure status and cardiac functional class*** |  |  |  |  |
| Type of heart failure |  |  |  | 0.426 |
| *Right-sided HF* | *51 (2.5)* | *51 (2.6)* | *0 (0.0)* |  |
| *Left-sided HF* | *477 (23.8)* | *466 (23.9)* | *11 (19.3)* |  |
| *Both-sided HF* | *1480 (73.7)* | *1434 (73.5)* | *46 (80.7)* |  |
| NYHA functional class |  |  |  | 0.005 |
| *Class II* | *353 (17.6)* | *344 (17.6)* | *9 (15.8)* |  |
| *Class III* | *1039 (51.7)* | *1021 (52.3)* | *18 (31.6)* |  |
| *Class IV* | *616 (30.7)* | *586 (30.0)* | *30 (52.6)* |  |
| Killip class |  |  |  | <0.001 |
| *Class I* | *527 (26.2)* | *523 (26.8)* | *4 (7.0)* |  |
| *Class II* | *1029 (51.2)* | *1013 (51.9)* | *16 (28.1)* |  |
| *Class III* | *392 (19.5)* | *371 (19.0)* | *21 (36.8)* |  |
| *Class IV* | *60 (3.0)* | *44 (2.3)* | *16 (28.1)* |  |
| ***Medical history and comorbidities*** |  |  |  |  |
| Previous MI, n (%) | 143 (7.1) | 139 (7.1) | 4 (7.0) | 1.000 |
| History of CHF, n (%) | 1872 (93.2) | 1828 (93.7) | 44 (77.2) | <0.001 |
| PVD, n (%) | 101 (5.0) | 98 (5.0) | 3 (5.3) | 0.762 |
| CeVD, n (%) | 150 (7.5) | 144 (7.4) | 6 (10.5) | 0.314 |
| Dementia, n (%) | 115 (5.7) | 114 (5.8) | 1 (1.8) | 0.255 |
| COPD, n (%) | 233 (11.6) | 229 (11.7) | 4 (7.0) | 0.273 |
| CTD, n (%) | 4 (0.2) | 4 (0.2) | 0 (0.0) | 1.000 |
| PUD, n (%) | 45 (2.2) | 43 (2.2) | 2 (3.5) | 0.368 |
| DM, n (%) | 466 (23.2) | 453 (23.2) | 13 (22.8) | 0.942 |
| CKD, n (%) | 475 (23.7) | 451 (23.1) | 24 (42.1) | <0.001 |
| Hemiplegia, n (%) | 12 (0.6) | 12 (0.6) | 0 (0.0) | 1.000 |
| Leukemia, n (%) | 0 (0.0) | 0 (0.0) | 0 (0.0) | NA |
| Lymphoma, n (%) | 1 (0.0) | 1 (0.1) | 0 (0.0) | 1.000 |
| Solid tumor, n (%) | 39 (1.9) | 37 (1.9) | 2 (3.5) | 0.304 |
| Liver disease, n (%) | 84 (4.2) | 75 (3.8) | 9 (15.8) | <0.001 |
| Charlson comorbidity index | 2.00 (1.00, 2.00) | 2.00 (1.00, 2.00) | 2.00 (1.00, 3.00) | 0.503 |
| ARF, n (%) | 7 (0.3) | 5 (0.3) | 2 (3.5) | 0.015 |
| ***Renal function markers*** |  |  |  |  |
| SCr, umol/L | 87.4 (65.1, 122.8) | 86.8 (64.7, 121.5) | 123.1 (81.3, 235.8) | <0.001 |
| Urea, mmol/L | 8.05 (5.91, 11.5) | 7.99 (5.89, 11.3) | 12.0 (7.36, 21.1) | <0.001 |
| UA, umol/L | 458.0 (361.0, 572.0) | 456.0 (360.0, 570.0) | 564.0 (403.0, 717.0) | <0.001 |
| eGFR, mL/min/1.73 m2 | 65.3 (42.3, 89.6) | 65.6 (43.1, 90.1) | 38.9 (17.7, 69.6) | <0.001 |
| CysC, mg/L | 1.56 (1.22, 2.20) | 1.55 (1.21, 2.18) | 1.97 (1.53, 4.08) | <0.001 |
| ***Complete blood count parameters*** |  |  |  |  |
| WBC, 10^9/L | 6.51 (5.09, 8.66) | 6.49 (5.06, 8.59) | 7.94 (6.01, 10.4) | <0.001 |
| MON percentage | 0.067 (0.052, 0.082) | 0.066 (0.052, 0.083) | 0.068 (0.052, 0.076) | 0.619 |
| MON count, 10^9/L | 0.420 (0.320, 0.570) | 0.420 (0.320, 0.570) | 0.480 (0.370, 0.680) | 0.018 |
| RBC, 10^12/L | 3.89 (3.44, 4.31) | 3.89 (3.44, 4.31) | 3.80 (3.00, 4.29) | 0.141 |
| RDW-CV, % | 14.4 (13.6, 15.6) | 14.4 (13.6, 15.6) | 14.7 (13.6, 15.9) | 0.460 |
| RDW-SD, fL | 47.8 (45.2, 51.4) | 47.8 (45.2, 51.4) | 48.9 (45.4, 51.4) | 0.355 |
| MCV, fL | 93.3 (88.8, 96.9) | 93.2 (88.8, 96.9) | 93.5 (89.5, 96.7) | 0.863 |
| HCT | 0.359 (0.316, 0.397) | 0.359 (0.316, 0.397) | 0.342 (0.276, 0.401) | 0.156 |
| LYM count, 10^9/L | 0.940 (0.620, 1.29) | 0.940 (0.630, 1.29) | 0.900 (0.480, 1.21) | 0.130 |
| MCH, pg | 30.5 (28.7, 32.0) | 30.6 (28.7, 32.0) | 30.1 (28.2, 32.0) | 0.367 |
| MCHC, g/L | 326.0 (318.0, 334.0) | 326.0 (319.0, 334.0) | 320.0 (311.0, 331.0) | 0.015 |
| MPV, fL | 12.1 (10.9, 13.2) | 12.1 (10.9, 13.2) | 11.7 (10.3, 13.1) | 0.163 |
| BASO count, 10^9/L | 0.030 (0.020, 0.040) | 0.030 (0.020, 0.040) | 0.020 (0.010, 0.030) | 0.075 |
| EOS count, 10^9/L | 0.060 (0.020, 0.140) | 0.060 (0.020, 0.140) | 0.030 (0.010, 0.080) | 0.001 |
| Hb, g/L | 117.0 (101.0, 131.0) | 117.3 (101.0, 131.0) | 112.0 (91.0, 133.0) | 0.124 |
| PLT, 10^9/L | 135.0 (101.0, 177.0) | 135.0 (101.0, 176.0) | 155.0 (107.0, 205.7) | 0.070 |
| PDW, % | 16.3 (16.0, 16.6) | 16.3 (16.0, 16.6) | 16.3 (15.8, 16.6) | 0.321 |
| Plateletcrit | 0.166 (0.131, 0.204) | 0.165 (0.131, 0.204) | 0.176 (0.132, 0.227) | 0.266 |
| NEUT count, 10^9/L | 4.84 (3.61, 6.80) | 4.82 (3.59, 6.75) | 6.09 (4.71, 9.27) | <0.001 |
| ***Coagulation parameters*** |  |  |  |  |
| D-dimer, mg/L | 1.29 (0.820, 2.54) | 1.27 (0.810, 2.48) | 2.17 (1.13, 5.88) | <0.001 |
| INR | 1.21 (1.13, 1.36) | 1.21 (1.13, 1.35) | 1.36 (1.19, 1.60) | <0.001 |
| APTT, s | 34.2 (30.9, 38.2) | 34.1 (30.9, 38.1) | 35.7 (32.0, 40.3) | 0.025 |
| TT, s | 17.2 (16.3, 18.0) | 17.2 (16.3, 17.9) | 17.5 (16.7, 18.6) | 0.016 |
| PTA, % | 68.5 (56.6, 78.1) | 68.5 (56.8, 78.4) | 59.2 (42.8, 69.3) | <0.001 |
| PTR | 1.22 (1.13, 1.36) | 1.22 (1.13, 1.36) | 1.33 (1.18, 1.61) | <0.001 |
| FIB, g/L | 3.04 (2.50, 3.69) | 3.04 (2.50, 3.69) | 3.32 (2.66, 4.33) | 0.057 |
| ***Myocardial injury, electrolytes, and biochemical markers*** |  |  |  |  |
| hs-cTn | 0.058 (0.024, 0.131) | 0.056 (0.023, 0.128) | 0.129 (0.075, 0.222) | <0.001 |
| CO2CP, mmol/L | 23.9 (20.9, 26.7) | 24.0 (21.0, 26.7) | 21.8 (17.4, 24.0) | <0.001 |
| Ca, mmol/L | 2.29 (2.18, 2.40) | 2.29 (2.18, 2.40) | 2.24 (2.11, 2.33) | 0.013 |
| K, mmol/L | 3.88 (3.53, 4.33) | 3.87 (3.52, 4.31) | 4.38 (3.75, 5.01) | <0.001 |
| Cl, mmol/L | 102.6 (98.8, 105.9) | 102.6 (98.8, 105.9) | 101.6 (95.4, 105.5) | 0.082 |
| Na, mmol/L | 139.0 (136.0, 141.3) | 139.0 (136.0, 141.4) | 136.8 (133.4, 139.2) | <0.001 |
| CK-MB/CK ratio | 0.180 (0.120, 0.260) | 0.180 (0.120, 0.260) | 0.160 (0.100, 0.220) | 0.052 |
| HBDH/LDH ratio | 0.82 (0.77, 0.87) | 0.82 (0.77, 0.87) | 0.81 (0.71, 0.89) | 0.385 |
| HBDH, U/L | 192.0 (162.0, 235.0) | 190.7 (162.0, 234.0) | 221.0 (177.0, 319.0) | <0.001 |
| AST, U/L | 28.0 (20.0, 52.0) | 28.0 (20.0, 51.0) | 36.0 (22.0, 206.0) | 0.002 |
| CK, U/L | 98.0 (66.0, 154.5) | 97.0 (66.0, 153.0) | 110.0 (86.0, 229.0) | 0.001 |
| CK-MB, U/L | 16.7 (12.3, 22.6) | 16.6 (12.3, 22.5) | 18.9 (12.1, 34.7) | 0.104 |
| LDH, U/L | 236.0 (198.0, 293.5) | 235.0 (197.0, 290.0) | 284.0 (211.0, 444.0) | <0.001 |
| BNP, pg/mL | 769.2 (310.6, 1739.4) | 754.9 (304.3, 1718.5) | 1518.2 (743.8, 2972.0) | <0.001 |
| ***Liver function, proteins, and lipid profile*** |  |  |  |  |
| AFU, U/L | 19.0 (16.0, 22.2) | 19.0 (16.0, 22.2) | 18.8 (14.7, 20.9) | 0.479 |
| ALB, g/L | 36.7 (33.5, 39.7) | 36.8 (33.6, 39.7) | 35.7 (31.9, 37.3) | 0.005 |
| AGR | 1.30 (1.10, 1.50) | 1.30 (1.14, 1.50) | 1.21 (1.00, 1.40) | 0.003 |
| GGT, U/L | 41.0 (23.0, 76.0) | 41.0 (23.0, 76.0) | 59.0 (26.0, 86.0) | 0.110 |
| ALT, U/L | 22.0 (14.0, 41.0) | 22.0 (14.0, 40.0) | 26.0 (16.0, 131.0) | 0.010 |
| IBIL, umol/L | 11.8 (7.9, 17.1) | 11.8 (7.9, 17.1) | 13.5 (7.3, 19.6) | 0.480 |
| ALP, U/L | 81.0 (65.0, 102.0) | 80.0 (65.0, 101.0) | 97.0 (72.0, 117.3) | 0.001 |
| GLB, g/L | 27.9 (24.8, 31.3) | 27.9 (24.8, 31.2) | 28.7 (25.0, 33.0) | 0.056 |
| DBIL, umol/L | 6.6 (4.2, 10.8) | 6.6 (4.2, 10.7) | 9.4 (4.4, 14.7) | 0.055 |
| TBIL, umol/L | 18.6 (12.4, 27.9) | 18.5 (12.4, 27.8) | 22.3 (12.1, 31.1) | 0.322 |
| TBA, umol/L | 6.00 (3.12, 10.89) | 6.00 (3.16, 10.80) | 6.10 (2.90, 13.25) | 0.397 |
| TP, g/L | 64.7 (60.3, 69.2) | 64.8 (60.3, 69.1) | 64.1 (60.0, 69.7) | 0.609 |
| TC, mmol/L | 3.61 (3.02, 4.27) | 3.61 (3.02, 4.27) | 3.60 (2.77, 4.19) | 0.229 |
| LDL-C, mmol/L | 1.77 (1.36, 2.25) | 1.77 (1.36, 2.25) | 1.67 (1.36, 2.15) | 0.693 |
| TG, mmol/L | 0.98 (0.72, 1.34) | 0.98 (0.72, 1.34) | 1.06 (0.77, 1.24) | 0.976 |
| HDL-C, mmol/L | 1.08 (0.87, 1.29) | 1.08 (0.87, 1.29) | 1.01 (0.78, 1.25) | 0.136 |
| ***Immune-inflammatory indices*** |  |  |  |  |
| PIV | 290.1 (150.2, 612.1) | 286.0 (147.8, 591.2) | 664.2 (253.9, 1041.1) | <0.001 |
| SIRI | 2.21 (1.21, 3.96) | 2.18 (1.20, 3.89) | 3.99 (2.26, 7.74) | <0.001 |
| SII | 699.5 (411.9, 1301.6) | 694.0 (406.4, 1267.9) | 1394.1 (639.6, 2310.3) | <0.001 |

**Notes:** Continuous variables are presented as median (interquartile range), and categorical variables are presented as n (%). P values were calculated using the Mann-Whitney U test for continuous or ordinal variables and the chi-square test or Fisher exact test for categorical variables, as appropriate. Age was treated as an ordinal categorical variable according to decade-based age categories: 1 = 21-29 years, 2 = 29-39 years, 3 = 39-49 years, 4 = 49-59 years, 5 = 59-69 years, 6 = 69-79 years, 7 = 79-89 years, and 8 = 89-110 years. HF_type was classified as right-sided, left-sided, or both-sided heart failure according to the original database coding. Symptom-to-door time was retained as the original ordered category because exact time ranges were not available in the analysis dataset. Identifier variables and follow-up outcome variables were not included in this baseline table.

**Abbreviations:**

AFU, alpha-L-fucosidase; AGR, albumin-to-globulin ratio; ALB, albumin; ALP, alkaline phosphatase; ALT, alanine aminotransferase; AMI, acute myocardial infarction; APTT, activated partial thromboplastin time; ARF, acute renal failure; AST, aspartate aminotransferase; BASO, basophil; BMI, body mass index; BNP, B-type natriuretic peptide; Ca, calcium; CCI, Charlson comorbidity index; CeVD, cerebrovascular disease; CHF, congestive heart failure; CK, creatine kinase; CK-MB, creatine kinase-MB isoenzyme; CKD, chronic kidney disease; Cl, chloride; CO2CP, carbon dioxide combining power; COPD, chronic obstructive pulmonary disease; CTD, connective tissue disease; CysC, cystatin C; DBIL, direct bilirubin; DBP, diastolic blood pressure; DM, diabetes mellitus; eGFR, estimated glomerular filtration rate; EOS, eosinophil; FIB, fibrinogen; GGT, gamma-glutamyl transferase; GLB, globulin; Hb, hemoglobin; HBDH, hydroxybutyrate dehydrogenase; HCT, hematocrit; HDL-C, high-density lipoprotein cholesterol; HF, heart failure; HR, heart rate; hs-cTn, high-sensitivity cardiac troponin; IBIL, indirect bilirubin; INR, international normalized ratio; K, potassium; LDH, lactate dehydrogenase; LDL-C, low-density lipoprotein cholesterol; LYM, lymphocyte; MAP, mean arterial pressure; MCH, mean corpuscular hemoglobin; MCHC, mean corpuscular hemoglobin concentration; MCV, mean corpuscular volume; MI, myocardial infarction; MON, monocyte;

MPV, mean platelet volume; Na, sodium; NEUT, neutrophil; NYHA, New York Heart Association; PCT, plateletcrit; PDW, platelet distribution width; PIV, pan-immune-inflammation value; PLT, platelet; PTA, prothrombin activity; PTR, prothrombin time ratio; PUD, peptic ulcer disease; PVD, peripheral vascular disease; RBC, red blood cell; RDW-CV, red cell distribution width-coefficient of variation; RDW-SD, red cell distribution width-standard deviation; RR, respiratory rate; SBP, systolic blood pressure; SCr, serum creatinine; SII, systemic immune-inflammation index; SIRI, systemic inflammation response index; TBA, total bile acid; TBIL, total bilirubin; TC, total cholesterol; TG, triglyceride; TP, total protein; TT, thrombin time; UA, uric acid; WBC, white blood cell.

**Formulas for selected immune-inflammatory indices:** PIV = NEUT x PLT x MON / LYM; SIRI = NEUT x MON / LYM; SII = PLT x NEUT / LYM.
