# Supplementary material for: Incremental prognostic value of pan-immune-inflammation value in conservatively treated acute myocardial infarction: derivation and MIMIC-IV validation
Source: Front Cardiovasc Med. 2026 Sep 10;13:1935766. doi: 10.3389/fcvm.2026.1935766 (PMC13602858; doi:10.3389/fcvm.2026.1935766)
Supplement: Supplementary file 4 [file Table2.docx]

**Supplementary Table S2 Subgroup analysis of PIV and 6-month all-cause mortality in patients with acute myocardial infarction**

| **Subgroup** | **Level** | **No. of patients** | **No. of deaths** | **OR (95% CI)** | **P value** | **P for interaction** |
| --- | --- | --- | --- | --- | --- | --- |
| Age | <69 years | 546 | 15 | 1.64(0.97-2.76) | 0.066 | 0.968 |
| Age | ≥69 years | 1462 | 42 | 1.56(1.15-2.11) | 0.004 |  |
| Sex | Female | 1163 | 28 | 1.59(1.13-2.26) | 0.009 | 0.992 |
| Sex | Male | 845 | 29 | 1.53(1.03-2.28) | 0.036 |  |
| Killip class | I-II | 1556 | 20 | 1.24(0.79-1.95) | 0.349 | 0.103 |
| Killip class | III-IV | 452 | 37 | 1.83(1.31-2.54) | <0.001 |  |
| NYHA class | II | 353 | 9 | 1.18(0.58-2.43) | 0.643 | 0.312 |
| NYHA class | III-IV | 1655 | 48 | 1.63(1.23-2.15) | <0.001 |  |
| HF type | Unilateral HF | 528 | 11 | 1.23(0.65-2.33) | 0.533 | 0.526 |
| HF type | Both-sided HF | 1480 | 46 | 1.67(1.25-2.25) | <0.001 |  |
| Diabetes mellitus | No | 1542 | 44 | 1.63(1.20-2.20) | 0.002 | 0.431 |
| Diabetes mellitus | Yes | 466 | 13 | 1.25(0.72-2.18) | 0.421 |  |
| Chronic kidney disease | No | 1533 | 33 | 1.56(1.11-2.19) | 0.011 | 0.997 |
| Chronic kidney disease | Yes | 475 | 24 | 1.50(1.00-2.25) | 0.049 |  |

**Notes:** Odds ratios are presented per 1-SD increase in natural log-transformed PIV. Subgroup-specific associations were estimated using logistic regression models adjusted for age category, sex, Killip class, NYHA class, and Charlson comorbidity index, except for the corresponding stratification variable when applicable.

Interaction P values were derived by adding a multiplicative interaction term between PIV and each subgroup variable to the adjusted logistic regression model. Acute renal failure and previous myocardial infarction were not included as subgroup variables because of sparse events in one stratum.

**Abbreviations:** CI, confidence interval; HF, heart failure; NYHA, New York Heart Association; OR, odds ratio; PIV, pan-immune-inflammation value; SD, standard deviation.
