# Supplementary material for: Incremental prognostic value of pan-immune-inflammation value in conservatively treated acute myocardial infarction: derivation and MIMIC-IV validation
Source: Front Cardiovasc Med. 2026 Sep 10;13:1935766. doi: 10.3389/fcvm.2026.1935766 (PMC13602858; doi:10.3389/fcvm.2026.1935766)
Supplement: Supplementary file 5 [file Table3.docx]

**Supplementary Table S3 Sensitivity analyses using SIRI and SII as alternative immune-inflammatory indices**

**Part A SIRI and SII analyzed as continuous variables**

| **Index** | **Model** | **OR (95% CI)** | **P value** | **AIC** |
| --- | --- | --- | --- | --- |
| SIRI | Model 1 | 1.72 (1.35-2.18) | <0.001 | 503.9 |
| SIRI | Model 2 | 1.48 (1.15-1.90) | 0.002 | 452.1 |
| SIRI | Model 3 | 1.47 (1.13-1.91) | 0.004 | 430.8 |
| SII | Model 1 | 1.79 (1.39-2.30) | <0.001 | 502.6 |
| SII | Model 2 | 1.53 (1.17-1.98) | 0.002 | 451.4 |
| SII | Model 3 | 1.54 (1.17-2.01) | 0.002 | 429.4 |

**Part B Quartile analyses of SIRI and SII with trend tests**

| **Index** | **Quartile** | **OR (95% CI)** | **P value** | **P for trend** |
| --- | --- | --- | --- | --- |
| SIRI | Q2 vs Q1 | 3.82 (1.03-14.23) | 0.046 |  |
| SIRI | Q3 vs Q1 | 4.23 (1.17-15.26) | 0.027 |  |
| SIRI | Q4 vs Q1 | 7.93 (2.33-27.00) | <0.001 | <0.001 |
| SII | Q2 vs Q1 | 1.94 (0.67-5.64) | 0.221 |  |
| SII | Q3 vs Q1 | 1.99 (0.70-5.68) | 0.199 |  |
| SII | Q4 vs Q1 | 3.90 (1.53-9.97) | 0.004 | 0.002 |

**Notes:** Odds ratios in Part A are presented per 1-SD increase in natural log-transformed SIRI or SII. In Part B, SIRI and SII were categorized into quartiles, with Q1 as the reference group.

SIRI and SII were analyzed in separate models and were not included simultaneously to avoid multicollinearity. Part B used the fully adjusted Model 3.

Model 1 was unadjusted. Model 2 was adjusted for age category, sex, Killip class, NYHA class, symptom-to-door time, and HF type. Model 3 was further adjusted for Charlson comorbidity index, history of myocardial infarction, history of chronic heart failure, diabetes mellitus, chronic kidney disease, and acute renal failure.

P for trend was calculated by entering quartiles of SIRI or SII as an ordinal variable into the fully adjusted logistic regression model.

**Abbreviations:** AIC, Akaike information criterion; CI, confidence interval; HF, heart failure; NYHA, New York Heart Association; OR, odds ratio; SD, standard deviation; SII, systemic immune-inflammation index; SIRI, systemic inflammation response index.
