# Supplementary material for: Incremental prognostic value of pan-immune-inflammation value in conservatively treated acute myocardial infarction: derivation and MIMIC-IV validation
Source: Front Cardiovasc Med. 2026 Sep 10;13:1935766. doi: 10.3389/fcvm.2026.1935766 (PMC13602858; doi:10.3389/fcvm.2026.1935766)
Supplement: Supplementary file 6 [file Table4.docx]

**Supplementary Table S4 Outcome replacement sensitivity analyses using 28-day and 3-month all-cause mortality**

**Part A PIV analyzed as a continuous variable**

| **Outcome** | **Deaths / total** | **Model** | **OR (95% CI)** | **P value** | **AIC** |
| --- | --- | --- | --- | --- | --- |
| 28-day mortality | 37/2008 | Model 1 | 2.19 (1.62-2.96) | <0.001 | 347.9 |
| 28-day mortality | 37/2008 | Model 2 | 1.81 (1.31-2.51) | <0.001 | 285.5 |
| 28-day mortality | 37/2008 | Model 3 | 1.85 (1.32-2.61) | <0.001 | 274.6 |
| 3-month mortality | 42/2008 | Model 1 | 2.09 (1.57-2.78) | <0.001 | 387.3 |
| 3-month mortality | 42/2008 | Model 2 | 1.75 (1.29-2.38) | <0.001 | 325.1 |
| 3-month mortality | 42/2008 | Model 3 | 1.79 (1.30-2.47) | <0.001 | 308.9 |

**Part B PIV quartile analysis with trend tests**

| **Outcome** | **Deaths / total** | **PIV quartile** | **OR (95% CI)** | **P value** | **P for trend** |
| --- | --- | --- | --- | --- | --- |
| 28-day mortality | 37/2008 | Q2 vs Q1 | 4.33 (0.68-27.37) | 0.119 |  |
| 28-day mortality | 37/2008 | Q3 vs Q1 | 3.27 (0.52-20.63) | 0.207 |  |
| 28-day mortality | 37/2008 | Q4 vs Q1 | 11.02 (2.02-60.02) | 0.006 | <0.001 |
| 3-month mortality | 42/2008 | Q2 vs Q1 | 6.10 (1.04-35.89) | 0.046 |  |
| 3-month mortality | 42/2008 | Q3 vs Q1 | 4.11 (0.69-24.49) | 0.120 |  |
| 3-month mortality | 42/2008 | Q4 vs Q1 | 12.66 (2.41-66.55) | 0.003 | <0.001 |

**Notes:** Odds ratios in Part A are presented per 1-SD increase in natural log-transformed PIV. In Part B, PIV was categorized into quartiles, with Q1 as the reference group.

Part B used the fully adjusted Model 3. Model 1 was unadjusted. Model 2 was adjusted for age category, sex, Killip class, NYHA class, symptom-to-door time, and HF type. Model 3 was further adjusted for Charlson comorbidity index, history of myocardial infarction, history of chronic heart failure, diabetes mellitus, chronic kidney disease, and acute renal failure.

P for trend was calculated by entering PIV quartiles as an ordinal variable into the fully adjusted model. Because 28-day and 3-month mortality had relatively few events, these analyses should be interpreted as sensitivity analyses.

**Abbreviations:** AIC, Akaike information criterion; CI, confidence interval; HF, heart failure; NYHA, New York Heart Association; OR, odds ratio; PIV, pan-immune-inflammation value; SD, standard deviation.
