# Supplementary material for: Incremental prognostic value of pan-immune-inflammation value in conservatively treated acute myocardial infarction: derivation and MIMIC-IV validation
Source: Front Cardiovasc Med. 2026 Sep 10;13:1935766. doi: 10.3389/fcvm.2026.1935766 (PMC13602858; doi:10.3389/fcvm.2026.1935766)
Supplement: Supplementary file 7 [file Table5.docx]

**Supplementary Table S5 Correlation and multicollinearity diagnostics**

**S5A Spearman correlation matrix among immune-inflammatory indices**

| **Index** | **PIV** | **SIRI** | **SII** |
| --- | --- | --- | --- |
| PIV | 1.000 | 0.908 | 0.884 |
| SIRI | 0.908 | 1.000 | 0.777 |
| SII | 0.884 | 0.777 | 1.000 |

**S5B Variance inflation factors in the fully adjusted PIV model**

| **Variable** | **VIF** | **Interpretation** |
| --- | --- | --- |
| HF type: left vs right | 7.94 | Moderate |
| HF type: both-sided vs right | 7.97 | Moderate |
| PIV, log-transformed | 1.04 | Low |
| Age | 1.06 | Low |
| Sex | 1.05 | Low |
| Killip | 1.09 | Low |
| NYHA | 1.12 | Low |
| Symptom-to-door time | 1.02 | Low |
| CCI | 2.60 | Acceptable |
| History of myocardial infarction | 1.20 | Low |
| History of chronic heart failure | 1.22 | Low |
| Diabetes mellitus | 1.50 | Low |
| Chronic kidney disease | 1.56 | Low |
| Acute renal failure | 1.01 | Low |

**Notes:** Spearman correlation coefficients were calculated among PIV, SIRI, and SII. All pairwise correlations among different indices were statistically significant at P < 0.001.

Variance inflation factors were calculated for variables included in the fully adjusted PIV model. PIV was entered as a natural log-transformed and standardized variable. HF type was treated as a nominal categorical variable.

The high correlations among PIV, SIRI, and SII support the strategy of analyzing these immune-inflammatory indices in separate models rather than including them simultaneously. No meaningful multicollinearity was observed for PIV in the fully adjusted model.

**Abbreviations:** HF, heart failure; PIV, pan-immune-inflammation value; SII, systemic immune-inflammation index; SIRI, systemic inflammation response index; VIF, variance inflation factor.
