# Supplementary material for: Incremental prognostic value of pan-immune-inflammation value in conservatively treated acute myocardial infarction: derivation and MIMIC-IV validation
Source: Front Cardiovasc Med. 2026 Sep 10;13:1935766. doi: 10.3389/fcvm.2026.1935766 (PMC13602858; doi:10.3389/fcvm.2026.1935766)
Supplement: Supplementary file 8 [file Table6.docx]

**Supplementary Table S6 Baseline characteristics of the MIMIC-IV external validation cohort according to 6-month all-cause mortality**

| **Characteristic** | **Overall (n=2679)** | **Survivors (n=1906)** | **Non-survivors (n=773)** | **P value** |
| --- | --- | --- | --- | --- |
| Age, years | 72.0 (62.0, 81.0) | 70.0 (60.0, 79.0) | 77.0 (67.0, 85.0) | <0.001 |
| Male sex | 1583 (59.1) | 1151 (60.4) | 432 (55.9) | 0.035 |
| Race |  |  |  | 0.206 |
| White | 1815 (67.7) | 1298 (68.1) | 517 (66.9) |  |
| Unknown | 442 (16.5) | 306 (16.1) | 136 (17.6) |  |
| Black | 204 (7.6) | 136 (7.1) | 68 (8.8) |  |
| Other | 86 (3.2) | 67 (3.5) | 19 (2.5) |  |
| Hispanic/Latino | 84 (3.1) | 66 (3.5) | 18 (2.3) |  |
| Asian | 48 (1.8) | 33 (1.7) | 15 (1.9) |  |
| Insurance |  |  |  | <0.001 |
| Medicare | 1460 (54.5) | 967 (50.7) | 493 (63.8) |  |
| Other | 1094 (40.8) | 838 (44.0) | 256 (33.1) |  |
| Medicaid | 125 (4.7) | 101 (5.3) | 24 (3.1) |  |
| Admission type |  |  |  | <0.001 |
| Emergency | 1134 (42.3) | 752 (39.5) | 382 (49.4) |  |
| Urgent | 1102 (41.1) | 822 (43.1) | 280 (36.2) |  |
| Observation | 420 (15.7) | 316 (16.6) | 104 (13.5) |  |
| Elective/Other | 23 (0.9) | 16 (0.8) | 7 (0.9) |  |
| ICU admission | 1937 (72.3) | 1290 (67.7) | 647 (83.7) | <0.001 |
| Charlson comorbidity index | 7.0 (5.0, 9.0) | 7.0 (5.0, 8.0) | 8.0 (7.0, 11.0) | <0.001 |
| Chronic heart failure | 1339 (50.0) | 897 (47.1) | 442 (57.2) | <0.001 |
| Diabetes mellitus | 1070 (39.9) | 741 (38.9) | 329 (42.6) | 0.085 |
| Chronic kidney disease | 819 (30.6) | 513 (26.9) | 306 (39.6) | <0.001 |
| Acute renal failure | 1120 (41.8) | 645 (33.8) | 475 (61.4) | <0.001 |
| History of myocardial infarction code | 272 (10.2) | 179 (9.4) | 93 (12.0) | 0.048 |
| White blood cell count | 11.0 (8.0, 15.2) | 10.5 (7.8, 14.2) | 12.6 (8.6, 17.6) | <0.001 |
| Neutrophil count | 8.74 (5.75, 12.77) | 8.27 (5.54, 11.87) | 10.39 (6.72, 15.06) | <0.001 |
| Lymphocyte count | 1.23 (0.77, 1.85) | 1.32 (0.84, 1.97) | 0.99 (0.64, 1.54) | <0.001 |
| Monocyte count | 0.59 (0.39, 0.90) | 0.58 (0.39, 0.86) | 0.64 (0.39, 1.00) | 0.002 |
| Platelet count | 210.0 (161.0, 272.0) | 213.0 (167.2, 271.0) | 200.0 (137.0, 275.0) | <0.001 |
| Albumin, g/L | 34.0 (29.0, 38.0) | 35.0 (31.0, 39.0) | 31.0 (26.0, 35.0) | <0.001 |
| PIV | 830.1 (354.6, 2009.7) | 720.7 (325.4, 1709.4) | 1239.8 (487.0, 3021.2) | <0.001 |
| SIRI | 4.12 (1.85, 9.51) | 3.36 (1.62, 7.79) | 6.62 (2.85, 14.94) | <0.001 |
| SII | 1448.8 (716.5, 2932.9) | 1296.6 (680.0, 2547.3) | 1961.4 (968.6, 3984.9) | <0.001 |

**Notes:** Data are presented as median (interquartile range) for continuous variables and n (%) for categorical variables. Analyses were performed in patients with available baseline PIV values. P values were calculated using the Mann–Whitney U test or chi-square test, as appropriate. AMI, acute myocardial infarction; CCI, Charlson comorbidity index; ICU, intensive care unit; PIV, pan-immune-inflammation value; SII, systemic immune-inflammation index; SIRI, systemic inflammation response index.
