# Supplementary material for: Incremental prognostic value of pan-immune-inflammation value in conservatively treated acute myocardial infarction: derivation and MIMIC-IV validation
Source: Front Cardiovasc Med. 2026 Sep 10;13:1935766. doi: 10.3389/fcvm.2026.1935766 (PMC13602858; doi:10.3389/fcvm.2026.1935766)
Supplement: Supplementary file 9 [file Table7.docx]

**Supplementary Table S7 Outcome replacement sensitivity analyses in the MIMIC-IV external validation cohort**

| **Outcome** | **Index** | **n** | **events** | **OR (95% CI)** | **P value** | **AIC** |
| --- | --- | --- | --- | --- | --- | --- |
| 28-day mortality | PIV | 2679 | 477 | 1.21 (1.08–1.34) | <0.001 | 2216.1 |
| 28-day mortality | SIRI | 2679 | 477 | 1.31 (1.17–1.46) | <0.001 | 2205.2 |
| 28-day mortality | SII | 2679 | 477 | 1.21 (1.09–1.35) | <0.001 | 2215.8 |
| 3-month mortality | PIV | 2679 | 661 | 1.16 (1.05–1.28) | 0.002 | 2581.5 |
| 3-month mortality | SIRI | 2679 | 661 | 1.26 (1.14–1.40) | <0.001 | 2568.8 |
| 3-month mortality | SII | 2679 | 661 | 1.16 (1.05–1.27) | 0.003 | 2582.0 |
| 6-month mortality | PIV | 2679 | 773 | 1.20 (1.10–1.32) | <0.001 | 2732.3 |
| 6-month mortality | SIRI | 2679 | 773 | 1.33 (1.20–1.46) | <0.001 | 2713.6 |
| 6-month mortality | SII | 2679 | 773 | 1.17 (1.06–1.28) | 0.001 | 2737.1 |
| 1-year mortality | PIV | 2679 | 920 | 1.14 (1.05–1.25) | 0.003 | 2913.2 |
| 1-year mortality | SIRI | 2679 | 920 | 1.25 (1.14–1.37) | <0.001 | 2899.6 |
| 1-year mortality | SII | 2679 | 920 | 1.10 (1.01–1.21) | 0.034 | 2917.4 |

**Notes:** ORs were estimated using fully adjusted Model 3 and are shown per 1-SD increase in natural log-transformed PIV, SIRI, or SII. Model 3 adjusted for age, sex, race, admission type, ICU admission, insurance, Charlson comorbidity index, chronic heart failure, diabetes mellitus, chronic kidney disease, acute renal failure, and history of myocardial infarction code. PIV, pan-immune-inflammation value; SII, systemic immune-inflammation index; SIRI, systemic inflammation response index.
